# Investigating pathways to environmental civic engagement for diverse communities

**DOI:** 10.1007/s00267-025-02356-2

**Published:** 2026-01-07

**Authors:** Aida Bagheri Hamaneh, Ashley A. Dayer, Tiffany A. Drape, Willandia A. Chaves

**Affiliations:** 1 Department of Fish and Wildlife Conservation, College of Natural Resources and Environment, Virginia Polytechnic Institute and State University, Blacksburg, VA USA; 2 Department of Agriculture, Leadership, and Community Education, College of Agriculture and Life Sciences, Virginia Polytechnic Institute and State University, Blacksburg, Virginia USA

**Keywords:** Asset-based framework, Environmental citizenship, Community cultural wealth

## Abstract

Environmental civic engagement provides an essential avenue to combat global environmental crises. However, opportunity and ability to participate in such civic action are not equal for everyone. Concerningly, the conservation movement in the U.S. has historically marginalized Black, Asian, and Latine voices from policy and decision-making processes. While previous research has focused on predictors of civic engagement in general and what barriers reduce participation, using an asset-based framing to consider what supports environmental civic engagement is less common. We used qualitative and quantitative methods to investigate the role of community cultural wealth (CCW) in Black, Asian, and Latine/Hispanic individuals’ participation in environmental civic engagement. CCW is an asset-based model, which has been used to understand persistence of marginalized groups in historically exclusionary spaces. We used CCW to identify factors that support Black, Asian, and Latine/Hispanic individuals’ environmental civic engagement as these behaviors have been affected by structural racism. Results showed that understanding systems of oppression and being motivated to change such systems were important predictors of environmental civic engagement for Black, Asian, and Latine/Hispanic individuals. Furthermore, our results suggest that organizations, social connections, and family connections are important sources of civic knowledge and opportunity. These findings suggest that taking an asset-based approach can be a promising way to support environmental civic engagement among Black, Asian, and Latine/Hispanic individuals.

## Introduction

### Environmental civic engagement

Researchers increasingly emphasize the role civic engagement plays in combating global environmental challenges like climate change, deforestation, and biodiversity loss (Brulle 2010; Ardoin et al. 2023). Civic engagement not only has the potential to combat global environmental issues but also provides benefits at the individual and collective levels (e.g., improved development in youth, increased community resiliency) (Ardoin et al. 2023; Brady et al. 2020). Civic engagement also allows a wider portion of society to have a say in how the world should look. In conservation, this not only contributes to a more equitable society, but it also opens avenues for diverse perspectives crucial for addressing complex problems (Naiman et al. 2019).

Concerningly, Black, Asian/Pacific Islander (referred to as “Asian” hereafter), and Latine/Hispanic individuals in the U.S. have historically been disenfranchised from environmental policy and decision-making processes (Finney 2014; Taylor 2016). These practices have lasting effects on these communities and their participation in environmental civic engagement (Finney 2014; Taylor 2016). For example, stereotypes that environmentalism is ‘White’ persist, despite higher environmental concern among many non-White groups (Pearson et al. 2018; Ballew et al. 2020). These perceptions can discourage Black, Asian, and Latine/Hispanic engagement with advocacy organizations, and impact collective action and policy decisions (Pearson et al. 2018).

Historically, U.S. environmental policy has favored White, affluent communities while neglecting concerns of ethno-racial minorities, leading to disproportionate burdens and exclusion from decision-making (Mohai et al. 2009). These burdens include proximity to hazardous waste facilities, reduced air quality, and reduced access to green spaces (Mohai et al. 2009; Finney 2014; Pearson et al. 2018). Strengthening civic engagement among Black, Asian, and Latine/Hispanic communities can help disrupt this cycle, address environmental injustices, and build broader support for conservation (Adler and Goggin 2005; Ardoin et al. 2023).

This study aimed to examine how Community Cultural Wealth (CCW; defined below) influences environmental civic engagement among Black, Asian, and Latine/Hispanic individuals. While previous research has examined environmental civic engagement among these groups, much of it has highlighted barriers to participation (e.g., Pearson et al. 2018; Finney 2014). Concomitantly, other research has emphasized youth agency and empowerment (e.g., Trott 2019; Ballard et al. 2017). We extend the latter by applying an asset-based framework, CCW, to environmental civic engagement, highlighting the resources that sustain participation.

Although there is no consensus on a definition of civic engagement (Serrat et al. 2022; Gibson 2000), it has been broadly defined as community service (Diller 2001), collective action (Adler and Goggin 2005), and political involvement (Diller 2001; Pickard 2022). We adapted Adler and Goggin’s definition and defined environmental civic engagement as ways people are involved in environmental decision-making processes in their communities to improve environmental conditions for themselves and others (Adler and Goggin 2005). This includes but is not limited to regular voting, advocating, contacting officials, or protesting about environmental issues (Keeter et al. 2002).

Environmental civic engagement falls under the umbrella of pro-environmental behaviors— behaviors that benefit or reduce harm to the environment (Larson et al. 2015; Ardoin et al. 2023). We were interested in environmental civic engagement rather than other pro-environmental behaviors, such as conservation lifestyle (e.g., recycling), because of the potential for civic engagement to provide agency for people in environmental decision-making in their communities (Adler and Goggin 2005). Previous research has examined predictors of civic engagement across various contexts, including the general public and marginalized communities, and within environmental issues. For example, Wray-Lake and Abrams’ (2020) study on urban Youth of Color revealed that safe community spaces, connecting with people with similar identities and interests, positive neighborhood and social connections, opportunities for local engagement, and positive adult interactions are related to civic engagement. Other studies on youth participation in civic engagement have linked political efficacy (i.e., perceived capacity to influence social and political change), parents, schools, community organizations, race and ethnicity, gender, and workplaces to civic engagement (Flanagan and Levine 2010; Brady et al. 2020; Wray-Lake and Abrams 2020). The present study expands on existing research to understand what supports environmental civic engagement among Black, Asian, and Latine/Hispanic individuals.

### Community cultural wealth

Community cultural wealth (CCW) is an asset-based model that seeks to understand strengths, skills, and knowledge that marginalized groups use to persist in exclusionary spaces (Yosso 2005). We adapted the CCW framework to understand factors that support environmental civic engagement for Black, Asian, and Latine/Hispanic individuals. This novel application of the framework is relevant given the historical disenfranchisement of these groups from the environmental movement.

CCW was developed to challenge deficit models, which asserted that Students of Color did not possess the cultural capital (e.g., an individual’s credentials, accent, or social etiquette) necessary to succeed in higher education (Yosso 2005). CCW posits that Students of Color possess a wealth of capital that is undervalued by the dominant culture, enabling them to succeed in exclusionary spaces despite challenges (Yosso 2005). Rather than focusing on what marginalized individuals lack, the model accentuates asset-based thinking to work towards equity in education (Yosso 2005; Denton et al. 2020). CCW conceptualizes this wealth in six forms of capital. For this study, we considered the following four because we believe they may be most relevant to environmental civic engagement: social capital, familial capital, navigational capital, and resistant capital (Table 1).

**Table 1:** The four types of capital from the community cultural wealth (CCW) framework used in this study

Capital	Definition (Adapted from Yosso, 2005)
Social capital	The support from networks of people and community resources
Familial capital	Cultural knowledge and emotional strength drawn from family ties and community traditions
Navigational capital	The skills, knowledge, and competencies needed to maneuver through inequitable systems
Resistant capital	An understanding of the structures of inequality and the motivation, skills and knowledge used to challenge that inequality

Research using the CCW framework focuses primarily on understanding how marginalized groups (e.g., Students of Color, first-generation students) persist in higher education (Denton et al. 2020; O’Shea 2016). CCW was used to explore experiences of first-generation college students in Australia (O’Shea 2016) and how Mexican American doctoral students accessed and persisted in graduate school (Espino 2014). In their review of CCW, Denton et al. (2020) describe the emotional and instrumental support provided by families, peers, student organizations, and faculty (i.e., social and familial capital). Often, this support allowed students to better maneuver through social institutions (i.e., navigational capital) (Denton et al. 2020). This illustrates a key point of CCW—that these forms of capital overlap (Yosso 2005; Denton et al. 2020). Another key finding of this review is that there is a lack of quantitative research on CCW (Denton et al. 2020), a gap that this study attempted to address.

While previous research has made significant progress in theorizing community engagement, much of it has focused on educational interventions (Flanagan and Levine 2010, Ardoin et al. 2023; Rabe 2024), civic/citizen science (Ballard et al. 2017), or political mobilization and activism (Adler and Goggin 2005; Firinci Orman and Demiral 2023). Asset-based frameworks, grounded on cultural strengths and community knowledge, remain relatively underutilized in environmental justice and civic engagement research. We build on emerging work linking cultural capital to civic engagement. For instance, Dimick 2012 proposed a framework for community empowerment in environmental science classrooms that aligns with CCW’s emphasis on agency. Morales-Doyle (2017) introduced justice-centered science pedagogy for community development and social transformation through environmental justice issues. Nxumalo and Montes (2023) focused on anticolonial, place-based climate justice pedagogies grounded in learning with land and community.

CCW also aligns with theories of critical consciousness and political efficacy. Critical consciousness emphasizes how marginalized groups develop awareness of structural inequities and take collective action to challenge them (Diemer et al. 2021). Political efficacy highlights the belief that someone’s civic actions can influence decision-making (Kahne and Middaugh 2008). Integrating CCW (Yosso, 2005) with these perspectives expands civic engagement scholarship (Krasny and Tidball 2015), positioning CCW as a framework beyond persistence in education. To our knowledge, this is one of the first studies to apply CCW in the context of environmental civic engagement research. Here, we examine how familial, social, navigational, and resistant capitals support environmental civic engagement among Black, Asian, and Latine/Hispanic individuals.

## Methods

The present study employed a multi-method approach (Dillman et al. 2014; Tashakkori and Teddlie 2003) to examine factors affecting participation of Black, Asian, and Latine/Hispanic individuals in environmental civic engagement. Latine or Hispanic refers to individuals whose cultural or national heritage originates from Latin America or Spanish-speaking countries. Since Indigenous environmental and civic engagement is guided by sovereignty, treaties, and specific relationships with land and water, it warrants a dedicated study; here, we focus on non-Indigenous populations and suggest future work designed in partnership with Indigenous communities (Whyte 2018; Norgaard, 2019). We conducted semi-structured interviews, which later informed the development of a nationwide survey. This study was conducted as part of a larger project on supporting nature-based recreation for Black, Asian, and Latine/Hispanic people. The Virginia Tech Institutional Review Board approved the project, recruitment materials, and data collection approach (Protocol # 22-113).

### Interview sampling

The qualitative portion of the study focused on Black, Asian, and Latine/Hispanic individuals between 18 and 25 years old who resided in the U.S. and participated in environmental civic engagement. We selected this age group because civic attitudes and skills formed in young adulthood continue to affect civic behavior throughout adulthood, making this an appropriate age group to facilitate civic engagement (Brady et al. 2020).

We employed convenience sampling by posting the study’s information to relevant social media accounts and email listservs. We conducted recruitment as part of a larger project focused on participation in nature-based outdoor activities (see Appendix 1 for recruitment materials). We targeted groups relevant to outdoor recreation (Finney 2014; Rowland-Shea et al., 2020) (e.g., Latino Outdoors, BIPOC Mountain Collective)—see Appendix 2 for a full list of groups. However, outdoor recreation and environmental civic engagement are often related (Wells and Lekies 2006). Although the importance of groups and organizations may have been overrepresented in our qualitative results, our findings can still provide insights into aspects of organizations that may be important. Because outdoor spaces can feel unwelcoming space for Black, Asian, and Latine/Hispanic individuals, and these groups are underrepresented in outdoor recreation (Outdoor Foundation, 2022), our interview sample may have missed people that do not participate in recreation but participate in environmental civic engagement. Findings need to be interpreted considering these sampling limitations.

We conducted 36 semi-structure interviews. Of these, 31 participants indicated participation in environmental civic engagement. Our interviews indicated that we had reached saturation within our data collection process because we began to hear much of the same information (Seidman 2006). See Appendices 3 and 4 for more details on the interview sampling. Anonymized demographic data for all participants is included in Appendix 5.

### Data collection using interviews

The lead author conducted all interviews virtually on Zoom. Interviews spanned about 45 to 60 min and consisted of open-ended questions (see Appendix 3 for details on data collection and Appendix 6 for interview questions). We defined environmental civic engagement broadly as “ways that you participate in your community or government in order to improve environmental conditions”, based on Adler and Goggin’s (2005) definition, and provided examples based on several indicators of civic engagement (Keeter et al. 2002). By communities, we refer to physical (e.g., neighborhoods, institutions such as churches, schools, or associations) and online communities (e.g., such as social media networks or affinity groups more specifically) with which participants have connections. With a broad definition of environmental civic engagement, we included sustained (e.g., volunteering, organizing) and more transactional forms (e.g., petitions, boycotts, “buycotts”). Although transactional activities are sometimes criticized as less substantive (Kristofferson et al. 2014), they represent important civic pathways for young people, who increasingly engage through digital platforms and consumption-based activism (O’Brien et al. 2018; Sloam 2022). This definition does not encompass all forms of environmental civic engagement. Thus, some other forms of engagement may have been missed—e.g., community monitoring (Conrad and Hilchey 2011), pollution-prevention initiatives (Meegoda et al. 2021), and non-normative youth “opt-out” actions (O’Brien et al. 2018). Our reasoning for using this definition was that such participation can have an influence on policy changes that affect historically marginalized groups.

We adapted questions from the significant life experience (SLE) approach to ask participants how they initially began participating and how they were able to continue participating (Arnold 2009). The SLE approach is a qualitative technique designed to uncover events and experiences that shaped an individual’s present environmental attitudes and behaviors (Chawla, 1998).

We asked participants about CCW by adapting Yosso’s (2005) definitions of the different forms of capital and by adapting interview questions from a dissertation (Gonzalez 2019) (Table 1). Our CCW questions asked interviewees about what encouraged their initial participation, what supported their continued participation, where they received information about environmental civic engagement, barriers to participation, and how they overcame those barriers.

### Analysis of interview data

The lead author conducted both inductive and deductive qualitative coding techniques in Dedoose. Deductive coding was based on the CCW framework (the four forms of capital) and our definition of environmental civic engagement to guide the thematic coding. Inductive coding was used to identify any other relevant themes beyond our deductive coding (Saldaña 2021). Regular peer debriefs with a project collaborator provided an external check for the coding process. In vivo coding, which uses participant’s own words as codes, was conducted to identify patterns and organize them systematically (Saldaña 2021). Each data segment could receive one or multiple codes. After open coding, focused coding was employed to refine initial categories into broader themes (Charmaz 2014). Focused codes grouped similar insights by comparing data points and highlighting connections between participants’ experiences. Axial coding was conducted as the final step, integrating categories and subcategories to one another to integrate the analysis and identify overarching themes (Corbin and Strauss, 2015). The codes included the four types of capital, in addition to related themes such as initial and continued participation in environmental civic engagement, environmental justice knowledge, experience with environmental degradation, knowledge of environmental issues, self-efficacy, and supporting future generations (Appendix 7 for code book). Our content analysis foregrounded meaning and context. Counts describe prevalence but do not determine salience (Saldaña 2021). For transparency, we report participant-level frequencies in Appendix 7. The quotes used in this paper are presented as spoken by participants, with some minor edits for clarity. To maintain readability, only the most illustrative quotes reflecting the themes or findings (Anderson 2010) are provided as examples.

### Survey sampling

The survey included individuals who were 18 years or older, including those who were not civically engaged in environmental issues. We used the QuestionPro Panel to recruit survey respondents. The audience panel is a pool of individuals who have enrolled to participate in panel surveys and receive a small compensation for their participation (Wardropper et al. 2021).

To recruit an appropriate sample, our survey screened for race (i.e., Black, Asian, or Latine/Hispanic), U.S. residency, and age (i.e., 18 years or older). Additionally, we used quota sampling methods (Bernard 2018) to ensure that respondents had similar characteristics to those of our population of interest (Jones et al. 2021; U.S. Census Bureau 2022) and participated in environmental civic engagement at a variety of frequencies (i.e. from non-participants to participants at various frequency). See Appendix 3 for additional information about sampling approach and Appendix 8 for quality checks.

### Data collection using surveys

We constructed an anonymous web-based survey (Appendix 9), which QuestionPro distributed to their panel directly via email. The email included a link to the survey and invited panelists to participate.

#### Measures of community cultural wealth

We used findings from qualitative interviews, our understanding of Yosso’s forms of capital, and existing survey items to construct four separate scales to measure social, familial, navigational, and resistant capital (Table 1; Sablan 2019; Hiramori et al. 2024). Social capital questions were adapted from existing scales, as social capital is an established construct used in numerous fields (Cohen and Wills 1985). Respondents were prompted to indicate how much they agreed with statements related to these capitals (1= Do not agree, 2= Slightly agree, 3= Moderately agree, 4= Mostly agree, 5= Completely agree). Examples of questions included for each form of capital are presented in Table 2. See survey instrument for all questions (Appendix 9).

**Table 2:** Examples of questions that were part of a battery of statements for each construct (i.e., each capital), with a 5-point Likert scale from “do not agree” to “completely agree”

Capital	Example of statement
Social Capital	There is at least one person I know whose advice I really trust.
Familial Capital	I have role models in my family.
Navigational Capital	I have developed strategies to navigate difficult people and situations.
Resistant Capital	I want to create a more just or equitable society.

These constructs were adapted from previous research (Cohen and Wills 1985; Sablan 2019; Hiramori et al. 2024).

#### Measures of participation in environmental civic engagement

Environmental civic engagement questions were adapted from the Questionnaire for the National Youth Survey of Civic Engagement (Zukin et al. 2002) and using indicators of civic engagement identified in Keeter et al. (2002). We measured participation in environmental civic engagement in two ways: first by asking individuals how often they participated (i.e., general environmental civic engagement), then by asking if they participated in specific aspects of environmental civic engagement (i.e., protesting for environmental causes, fundraising for environmental organizations, etc.) within the last twelve months or five years. Our list included 15 different types of environmental civic engagement, and an additional open category (“other”). Thus, we believe our list included a broad range of engagements. However, these specific dimensions of environmental civic engagement are not exhaustive. Some forms of engagement may have been missed.

#### Measures of initial participation

To gauge initial participation, participants were prompted to select why they initially engaged in environmental civic engagement from a provided list, including options of a friend, family member, role model, or an organization. Subsequently, respondents indicated their level of agreement for each option using a 5-point Likert scale ranging from “strongly disagree” to “strongly agree”.

#### Measures of race and ethnicity and age

A single multi-select question evaluated both race and ethnicity (Krogstad and Cohn 2014). We then classified all participants into five distinct groups: individuals identifying as Latine/Hispanic of any race, those identifying as Black or African American alone, those identifying as Asian alone, and those identifying as Multiracial non-Latine/Hispanic. We also assessed participants’ age to compare young adults (18 to 25 years old) to older adults (>25 years old).

### Analyses of survey data

#### Exploratory Factor Analysis (EFA) of community cultural wealth

To determine the underlying structure of our CCW scale, we conducted separate EFA’s for social, familial, navigational, and resistant capital scales. Once we had the final factors, we determined Cronbach’s alpha. See Appendix 3 for more details on EFA analysis.

#### Factors associated with participation in environmental civic engagement

We weighted our data to match the U.S. census distribution of ethno-racial demographics (which was not completely accomplished by sampling quotas) using a bootstrapping method (Jones et al. 2021; US Census Bureau 2019; 2022). We standardized all continuous predictor variables to have a mean of zero and a standard deviation of one. No collinearity was observed among predictor variables. We conducted all analyses using the statistical software R Studio (v4.1.3; R Core Team 2023).

We used an ordered logistic regression (Lattin et al. 2003) to analyze predictors of general environmental civic engagement and the five most frequently identified forms of environmental civic engagement. Results for each predictor are marginal effects, after accounting for other factors in the model. For each variable, we calculated the likelihood of participating in environmental civic engagement using odds ratios with a statistical significance level of *α* = 0.1.

## Results

### Qualitative results

#### Social capital

When asked about participation in environmental civic engagement, most interviewees discussed how their social capital affected both their initial and continued involvement. Social capital resulted from several sources, including community organizations, school organizations and clubs, educators, social media, and friends. These sources of social capital facilitated participation by providing emotional support (e.g., accompanying them to a protest) and instrumental support (e.g., providing information about environmental issues or opportunities to engage). These social ties are interconnected with other forms of capital, providing belonging and encouragement (familial), and resources such as procedural knowledge (navigational).

Social capital supported initial participation in several ways. Some individuals described meeting passionate friends or educators who introduced them to environmental civic engagement. For example, one person, who had knowledge of environmental issues, said that “it wasn’t fully until [they] met people who it was their life’s goal, passion, to really take care of their community, their outdoors” that they began to participate. This example, among other similar statements, suggests that while people may develop interest in environmental civic engagement by learning about environmental issues, participation was supported by social capital. Similarly, another interviewee said they would “probably still care” about environmental issues, but that “it definitely makes it easier to be active when your friends are”. Other people began participating because of organizations or institutions. For example, a participant described how their first experience with environmental civic engagement was through a tree planting initiative hosted by their local city. In many cases, interviewees reported that social capital facilitated initial and continued participation by providing information about environmental issues and opportunities to participate in environmental civic engagement. In other words, social capital led to navigational capital for many interviewees. For instance, this participant said, “when there [are] people directly around you who … are making it easy to be involved by … showing you where you can copy and paste… really facilitate that for me.”

In some cases, participants leveraged their social capital to increase their influence by sharing information with their social networks. For example, one person said they feel as if they have “a bigger impact” if they convince their friends to sign a petition with them. Another participant described how his local community was able to more efficiently enact environmental change and protect their local natural resources by using their network of community members for collective action. Taken together, social networks not only initiated and sustained participation but also led to navigational capital. When mobilized collectively, social capital was connected to resistant capital through shared commitment to environmental causes.

#### Familial capital

Familial capital manifested as support from family, extended family, and community, and contributed to the participation of most interviewees. While familial capital was mostly spoken of in terms of continued engagement, it did support initial participation of a few people (e.g., participants’ parents conveyed the importance of civic duty or of protecting the environment). Familial capital manifested as attentive audience (with relatives listening to participants’ experiences on environmental civic engagement), emotional support (as families offered encouragement and practical help), and intergenerational influence (when relatives followed participants’ advice). One interviewee said their family “understands their passion” for environmental civic engagement and that being close to family keeps them from being isolated. Similarly, interviewees emphasized the importance of community. One interviewee described how working in some spaces lends itself to community and how that community of like-minded people can be encouraging. When asked what supports her continued participation, she said, “I think knowing that there’s always a community of people that are working in this sector, that you’re never really alone”. Another interviewee described how connecting with their community by doing things like group dinners helped regenerate their drive for environmental change: “building a really strong, resilient community is one of the ways that I felt refreshed from having to constantly be exposed to all of the issues that are happening”. In this case, the participant and their friends built resilience by strengthening their community.

Familial capital often overlapped with other forms of capital. Familial support led to social capital by reinforcing belonging and mutual aid within community networks. When relatives followed participants’ advice, familial trust intersected with navigational capital to translate care into action.

Participants also framed their continued participation in terms of protecting conditions and resources for relatives and future generations (Zaval et al. 2015). For example, an interviewee whose extended family lived in the Southeast Asia expressed that they felt the need to act partially out of concern for them, given they are “more vulnerable to climate change impacts like sea rise”. Another person said they continue persisting because they want themselves and their younger nephews to “have a healthy environment”. Another woman spoke about how she wants to be a mother someday and how she’s “concerned about what conditions they’ll grow up in and how they’ll view the world” and so she contributes “to programs where [she] can help future generations, including the children in [her] family and [her] friends’ children.

#### Navigational capital

In almost all cases, interview participants reported that they used navigational capital to participate in environmental civic engagement. Navigational capital was most often spoken of in relation to continued participation. Participants primarily described these forms of navigational capital: (1) skills and information related to civic engagement processes and (2) information about opportunities to be civically engaged. Participants leveraged their social and familial capital to learn this essential information. Most often, participants indicated that they relied on community, organizations, colleges, social media, environmental education, and family for the necessary information and skills. These navigational resources frequently overlapped with familial capital (kin-based trust that made advice actionable) and social capital (networks that circulated opportunities).

Several interviewees attributed their knowledge of civic engagement processes to their social circles. One interviewee described how their friends would share information about public comment periods and opportunities to contact representatives, which “definitely increased” their level of engagement. As one interviewee put it, “having people in your life who are knowledgeable about how to fight against certain groups would be the key”. In another case, an interviewee recollected how their connections to a friend who works in policy helped them participate more effectively by using impactful techniques: “a lot of my friends that … work in policy … helped me … stay very privy to … what legislative [unknown] they listened to. So they’re like, …the emailing helps, the calling helps; the petitions don’t really help…’ so they give me more like … what actually works.” Here, social capital (policy-savvy friends) intersected with navigational capital (what to do).

Many participants stated their social connections provided information about opportunities to participate in environmental civic engagement. Several participants used social media for this purpose. As one interviewee put it, social media is “definitely helpful” because “[you’re] just one click away from signing a petition or one click away from getting a link where you can type two paragraphs about why they shouldn’t be drilling this pipeline…”. Others spoke about using connections to local organizations to learn more about local opportunities. One participant commented that they were “a lot more aware” of opportunities to participate because of their local community organizations. These examples illustrate overlap of social and navigational capitals. That is, networks expose opportunities while practical knowledge enables action.

Colleges emerged as an important source of opportunities for civic engagement. For example, this participant spoke about how going to college and connecting with different organizations like Latino Outdoors impacted her participation in environmental civic engagement:

If I hadn’t gone to college and if I hadn’t been a part of all these groups, I don’t know if I would have been really civically engaged. Because I wouldn’t have access to these different networks, I have access to now, and I wouldn’t have gone to these talks that tell you about how impactful it is—all these facts about public comments. I would probably feel really similar to how a lot of people in my community feel. I would probably feel really disempowered just seeing how things go for them all the time.

It seems that for this participant, connecting with different organizations and learning about the impacts of civic engagement through school provided her with political efficacy that she would not otherwise have, which was essential to her participation. This view that political efficacy is necessary for continued engagement was echoed by others. In these college-based accounts, institutional networks (social capital) supplied opportunities while training and campaign infrastructure provided navigational capital.

#### Resistant capital

Approximately half of interviewees reported using resistant capital to participate in environmental civic engagement. For these participants, resistant capital manifested in their understanding of systems of inequality and how they challenged that inequality to continue participating. Many participants expressed an understanding of structural racism within civic engagement processes. They spoke about feeling like their voices went unheard, about having higher barriers to participation because of their ethno-racial identities, and the belief that “the system helps some communities more than others”. Some individuals expressed understanding of institutional racism by speaking about tokenization and the lack of diversity within environmental organizations. For example, one interviewee said:

If you have only White managers on a team, you will never create a space of diversity because your diverse staff will probably feel uncomfortable saying things to you. Most big greens are mainly White…And diversity is not one person of color.

Other participants expressed an understanding of environmental justice and spoke on how their communities shoulder a higher burden of negative environmental impacts.

Despite exhibiting awareness about these inequities, interviewees continued to persevere in the civic engagement space. Several participants indicated that they were motivated to create change in these spaces for present and future generations. For instance, as this participant put it, “I think just seeing and advocating for the change … that more people can be involved, especially minorities. It’s important [for] me to try to do my part to advocate for change [with the] hope that everyone has equal access to their civic duties…” As for future generations, this interviewee said: “that desire to pave a path for the future generation is what inspires me, what motivates me to get involved in that kind of stuff. Because if an Asian kid can see me and feel inspired, that’s all I want”. Another person who had access to nature experiences as a kid was inspired to participate in civic engagement so other kids from the city could have the same opportunities.

Others persevered by creating spaces for Black, Indigenous, and people of color (BIPOC) within the civic engagement sphere. One interviewee alluded to this by saying, “if I can’t be authentic in a space, then I don’t want to be in that space. And sometimes it’s better to just create your own space”. Another participant indicated that being in diverse spaces and “having Black and Indigenous people in [their] life” provided them with knowledge about environmental justice that they would otherwise lack. While several interviewees expressed desire and motivation to enact “system-oriented” change, many of those same people indicated that they “wouldn’t even know where to start”.

#### Other factors

A few inductive themes emerged in our analysis. Several participants spoke about experiencing negative environmental effects as contributing to their engagement with civic activities. In addition, nearly half of the interviewees said they participated in environmental civic engagement because of their connection to nature. Some participated to get outside and interact with nature, as was the case with one person who volunteered for a reforestation project. Others expressed that it was their “love for nature” that led them to participate in civic engagement related to the environment.

Environmental Education (EE) was an explicit part of our qualitative scripts (Appendix 6). EE was cited as important for their initial and continued participation in environmental civic engagement. However, results related to EE would require an in-depth discussion, which is beyond the scope of this paper. Thus, we do not present the results of EE beyond the information included in code book (Appendix 7).

### Quantitative results

#### Exploratory factor analysis

We conducted an Exploratory Factor Analysis (EFA) with principal axis factoring for all 20 items within our CCW scale as part of our study on nature-based activities (Table S10.1 in Appendix 10). After removing low-scoring items, results revealed 18 items distributed across four factors. Notably, each factor corresponded directly to one of the four forms of capital: social (Cronbach’s *α* = 0.77), familial (Cronbach’s *α* = 0.89), navigational (Cronbach’s *α* = 0.81), and resistant (Cronbach’s *α* = 0.82) (Bagheri Hamaneh et al. (n.d.). We averaged the items within each factor to create composite measures for social, familial, navigational, and resistant capital.

#### Factors associated with participation in environmental civic engagement

Of the 1709 complete survey responses, 1387 were usable, and 322 were removed due to failed attention and quality checks (Wardropper et al. 2021). We then applied post-stratification weights to match U.S. Census ethno-racial distributions. These weights inflate underrepresented subgroups and deflate overrepresented ones, so the weighted total is 1808 surveys.

**Fig. 1:**
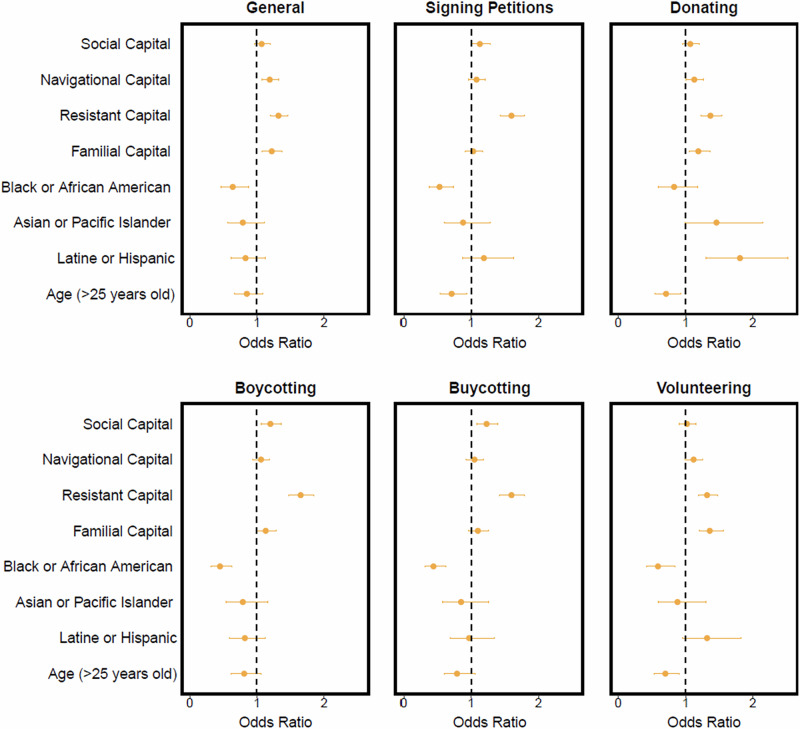
The relative effect of predictors on general participation in civic engagement and the five most frequently identified forms of environmental civic engagement activities. Error bars are the 95% confidence intervals (CI). CIs that cross the OR of one (i.e., the dashed line) indicate no significant effect

When asked about their participation in general environmental civic engagement within the past five years, approximately 67% of respondents indicated that they participated. Those who participated did so most often by boycotting companies and products because they cause environmental harm (*n* = 664), signing petitions to support environmental initiatives (*n* = 503), “buycotting” (i.e., buying a product because it’s environmentally friendly) (*n* = 481), donating to environmental causes or organizations (*n* = 467), and volunteering for environmental organizations (*n* = 344). When asked to rank (5-point Likert scale from do not agree to completely agree) how much a friend, family member, role model, or organization, group, or club had an effect their initial participation in civic engagement, respondents most often indicated that they first started participating because of an organization, group, or club. Further respondent characteristics are reported in Appendix 11 (Table S11.1). We found that resistant capital was associated with all our civic engagement measures (Fig. 1; and Tables S12.1–S12.6 in Appendix 12). For each increase in one standard deviation (SD) in resistant capital (equivalent to 0.98), the odds of participating in general civic engagement (Odds Ratio [OR] = 1.32; 95% Confidence Interval [CI] = 1.20–1.46), boycotting (OR = 1.65; =1.47–1.84), signing petitions (OR = 1.60; CI = 1.43–1.79]), donating (OR = 1.37; CI = 1.23–1.54), volunteering (OR = 1.32; CI = 1.19–1.48]), and “buycotting” (OR = 1.60; 1.42–1.80) also increased. We found that as familial capital increased by one SD (equivalent to 0.95), individuals were more likely to participate in general in civic engagement (OR = 1.22; CI = 1.08–1.37), donate (OR = 1.19; CI = 1.05–1.36), boycott (OR = 1.13; CI = 1.00–1.29), and volunteer (OR = 1.36; CI = 1.20–1.56). Familial capital was not associated with signing petitions and “buycotting”. For each SD increase in social capital (SD = 0.94), there was an increase the odds of boycotting (OR = 1.20; CI = 1.06–1.35), “buycotting” (OR = 1.23; CI = 1.08–1.40), and signing petitions (OR = 1.13; CI = 1.00–1.28). However, social capital was not associate with general civic engagement, volunteering, or donating. As navigational capital increased by one SD (equivalent to 0.95), the odds of general participation in civic engagement (OR = 1.19; CI = 1.07–1.32), donating (OR = 1.13; CI = 1.00–1.27), and volunteering (OR = 1.07; CI = 0.99, 1.26) increased. Navigational capital was not associated with the odds of participating in boycotting, signing petitions, or “buycotting” (Fig. 1; Appendix 12).

Results showed that survey respondents who identified as Black or African American had lower odds of general participation in environmental civic engagement (OR = 0.64; CI = 0.46–0.87), boycotting (OR = 0.45; CI = 0.32–0.63), “buycotting” (OR = 0.44; CI-0.31–0.63), volunteering (OR = 0.59; CI = 0.42–0.84), and signing petitions (OR = 0.53; CI = 0.38–0.74) than the baseline (i.e. multiracial non-Latine/Hispanic) (Fig. 1). Those who identified as Latine/Hispanic or Asian had higher odds of donating to environmental organizations when compared to multiracial non-Latine/Hispanic individuals (OR = 1.81; CI = 1.30–2.52 and OR = 1.46; CI = 0.99–2.15, respectively) (Fig. 1; Appendix 12). The odds of volunteering also increased for participants who identified as Latine/Hispanic (OR = 1.32; CI = 0.96–1.83) when compared to multiracial non-Latine/Hispanic (Fig. 1). We did not find an association with race for Asian or Latine/Hispanic respondents when we asked about participation in civic engagement in general. Age (i.e., ≤25 versus >25 years old) was not associated with general participation in environmental civic engagement, boycotting, and buycotting. Older adults were less likely to participate in volunteering (OR = 0.70; CI = 0.53–0.91), donating (OR = 0.71; CI = 0.54–0.93), and signing petitions (OR = 0.71; CI = 0.54–0.94) than younger adults.

## Discussion

We investigated the influence of community cultural wealth (CCW) in fostering participation in environmental civic engagement among Black, Asian, and Latine/Hispanic individuals. This novel use of the CCW framework showed that resistant, familial, social, and navigational capital were all positively associated with one or more measures of environmental civic engagement (i.e., general participation, boycotting, “buycotting”, volunteering, donating, and signing petitions). Notably, we demonstrated a significant relationship between resistant capital and all six measures of civic engagement. Qualitative findings further revealed some ways people leverage their capital to be civically engaged. Aligning with Yosso (2005), our qualitative results showed that these forms of capital often overlap, with social ties contributing to navigational resources, familial commitments influencing resistance capital, and navigational knowledge enabling social networks to translate concerns into action.

### Community cultural wealth and participation in environmental civic engagement

Quantitative findings showed that resistant capital was linked with higher odds of participating in all measures of civic engagement assessed in this study. This suggests that knowledge of structures of oppression and motivation to challenge those structures may be important for fostering environmental civic engagement among Black, Asian, and Latine/Hispanic individuals. These findings reflect earlier studies that link the knowledge of systemic injustices and critical reflection with civic action (Diemer et al 2021). Notably, we asked about knowledge of structures of oppression in general, and not specifically about knowledge of environmental injustice (i.e., inequitable distribution of environmental benefits and burdens). However, environmental and social justice issues may be inexorably linked (Ergas et al. 2021). For example, several interview respondents mentioned that experiencing environmental degradation contributed to their interest in environmental civic engagement, an emerging theme highlighting that social-environmental justice connection. Taken together, these findings suggest that considering environmental civic engagement through an asset-based lens, highlighting the interconnectedness of environmental and social issues, and addressing concerns specific to these communities could be essential for boosting participation. Our findings echo work showing that civic engagement strengthens when social and environmental issues are connected (Quiroz-Martinez et al. 2005; Flanagan et al. 2019). For instance, Flanagan et al. (2019) found that linking racial justice to environmental issues increased youth motivation to participate in civic activities.

Curiously, resistant capital was not salient for all interview participants, with about half of our interviews coded for it. This difference between qualitative and quantitative results may be due to the nature of our questions for each method - quantitative questions explicitly asked about resistant capital, whereas qualitative questions were more indirect. Of the interviewees who relied on resistant capital, several emphasized their perception of inequities within civic engagement processes, noting that certain groups wielded more influence than others. Others expressed concern about the predominantly White makeup of major environmental organizations. Interviewees persisted with their civic engagement, despite being aware of these disparities, in part because they were fueled by a determination to support future generations, like findings from another CCW study on Latine graduate healthcare students (Zell 2014). Others were driven by the mission to create inclusive spaces that amplify the voices of BIPOC communities. A notable subset of participants actively sought to instigate change. In fact, multiple interviewees underscored the critical role of systemic change in promoting diverse participation in environmental civic engagement and in advancing environmental justice initiatives.

The other forms of capital (i.e., navigational, social, and familial) were linked with one or more quantitative measures of environmental civic engagement, but not to all of them. For example, social capital was positively associated with boycotting, “buycotting”, and signing petitions. In the qualitative interviews, interviewees described deriving instrumental and social support from their social networks, including organizations and institutions. Some participants said that despite having a previous interest in civic engagement, it was their social capital that ultimately moved them to act—a finding that supports previous studies which link peer relationships with increased civic engagement (Barrett and Pachi 2019; Wray-Lake and Abrams 2020; Sloam 2022). Similarly, familial capital (which was linked to general civic engagement, volunteering, and donating in the quantitative analyses) provided essential emotional and instrumental support to the interviewees. These findings align with other studies that linked family political interest, political knowledge, values, and political behavior to young people’s knowledge and participation in civic engagement (Barrett and Pachi 2019; Flanagan and Levine, 2010; Wray-Lake and Adams, 2020). In addition to relatives, having a supportive community was regenerative for some interview participants and helped them persist with their civic actions. Familial capital also manifested to protect conditions and resources for relatives and future generations, which is consistent with generativity theory (McAdams and de St. Aubin, 1992; Zaval et al. 2015) and findings that legacy and parenthood concerns increase pro-environmental engagement (Hurlstone et al. 2020; Shrum et al. 2023).

Lastly, navigational capital was only associated with general environmental civic engagement. Interview participants spoke about how their social and familial capital led to navigational capital, by providing essential skills, information, and opportunities. This is consistent with political-efficacy pathways whereby learning how to navigate civic institutions is associated with higher participation (Kahne and Sporte 2008; O’Brien et al. 2018). Our finding underscores the importance of establishing pathways to cultivate social or familial capital. One potential avenue could be to connect with organizations like affinity groups and colleges, which many interview participants identified as influential. Several studies have demonstrated that institutions such as community organizations and colleges are linked to civic engagement (Barrett and Pachi 2019; Flanagan and Levine 2010; Flanagan and Bundick).

Some interviewees associated their increased knowledge and skills (i.e., navigational capital) with a strengthened sense of political self-efficacy. These participants highlighted the importance of political self-efficacy in environmental civic engagement, which is consistent with existing literature on political self-efficacy (Wray-Lake and Abrams 2020; Ardoin et al. 2023). Therefore, providing opportunities to learn more about civic engagement processes and skills could strengthen political self-efficacy and increase environmental civic engagement.

### Other factors associated with environmental civic engagement

While we observed variations in participation rates across ethno-racial groups and specific types of civic engagement (e.g., Latine/Hispanic and Asian respondents had higher odds of donating to environmental organizations), we did not find a difference between these ethno-racial groups for general environmental civic engagement. This suggests that while these groups may have different preferences for civic engagement types (or face diverse barriers), they may participate at similar rates. Offering a variety of opportunities can ensure that individuals from diverse ethno-racial backgrounds have civic engagement activities aligning with their preferences and abilities.

Black or African American respondents were less likely than Multiracial respondents to participate in the forms of civic engagement we measured. This may reflect lower participation in mainstream environmental organizations and activities, where leadership and membership remain disproportionately comprised by White people (Taylor 2014; Meaux et al. 2021). Concomitantly, Black communities have a strong record of leadership in environmental justice efforts such as pollution, toxic waste, and residential segregation (Bullard 1990; Taylor 2014; Brinkley et al. 2022). Although our measure of environmental civic engagement included several specific forms, it may not have captured the breadth of environmental action relevant to Black individuals.

Younger and older adults showed similar participation in general engagement, but younger adults were more likely to sign petitions and donate to environmental causes. Our results indicate that while age is not associated with participation in overall environmental civic engagement, the specific forms of engagement differ with age. This may reflect young adults’ greater use of online platforms, which facilitate low-cost, accessible forms of activism such as petitions and donations.

In the qualitative interviews, nearly half cited their “love for nature” as a reason to engage. This nature-civic link is well established in the literature (Wells and Lekies 2006; Kals et al. 1999; Larson et al. 2011). These findings suggest that nature interaction can support participation in environmental civic engagement for Black, Asian, and Latine/Hispanic individuals. These results underscore the significance of diversifying nature-based recreation, which has historically excluded these communities (Outdoor Foundation 2022; Rowland-Shea et al. 2020).

### Recommendations

In accordance with our resistant capital results, fostering an understanding of inequities within environmental civic engagement may be a key approach to garner environmental interest and action among Black, Asian, and Latine/Hispanic individuals. This could entail the integration of awareness of inequities into curricula and programming by environmental education programs that incorporate environmental justice perspectives, emphasizing the interconnection between environmental and racial issues, or framing lessons in a manner that reflect the lived experiences of different groups.

Additionally, ensuring that Black, Asian, and Latine/Hispanic individuals have institutional opportunities to participate in environmental civic engagement is important and leverage their social, familial, and navigational capital. Community-based organizations, faith-based organizations, and affinity groups are well positioned to provide accessible opportunities for engagement. Investments in these organizations can cultivate resilient networks that encourage participation in environmental civic engagement.

Finally, our results underscore the role higher education in building navigational capital. However, not all young adults have access to four-year institutions. To ensure access to civic opportunities, it is essential to provide alternative pathways outside higher education. Conservation organizations, community-based organizations, faith-based institutions, and digital platforms can all serve as places for environmental civic engagement. These venues can provide training, mentorship, and opportunities for advocacy that reach young people who might otherwise not be included in civic experiences.

This study expands Community Cultural Wealth into the domain of environmental civic engagement. By showing how cultural capital (social, navigational, familial, and resistant) is associated with distinct forms of engagement, this study positions CCW framework as a valuable lens for understanding participating in environmental civic engagement among Black, Asian, and Latine/Hispanic communities.

## Supplementary information

Appendix 1

Appendix 2

Appendix 3

Appendix 4

Appendix 5

Appendix 6

Appendix 7

Appendix 8

Appendix 9

Appendix 10

Appendix 11

Appendix 12

## Data Availability

Following the ethics approvals for this research, we will not make the data publicly available. Data will be made available upon request, provided necessary safeguards to protect participant confidentiality.
