# Supplementary material for: Investigating pathways to environmental civic engagement for diverse communities
Source: Environ Manage. 2026 Jan 7;76(2):61. doi: 10.1007/s00267-025-02356-2 (PMC12779674; doi:10.1007/s00267-025-02356-2)
Supplement: Supplementary file 1 — Appendix 1 [file 267_2025_2356_MOESM1_ESM.docx]

Appendix 1

*Interview Recruitment Materials*

*Email to organizations*

Hello,

I am a master's student at Virginia Tech conducting a research study to better understand how to support Black, Asian, and Latine folks' participation in outdoor recreation and environmental civic engagement (IRB Protocol #22-113). I am looking for potential participants to interview as part of this study. I would greatly appreciate it if you could forward this email to your [insert organization] email list to help with this effort.

Understanding what initiates and supports Black, Asian, and Latine people’s participation in outdoor recreation can help park managers, environmental educators, and other practitioners create more inclusive, supportive, and culturally relevant programs. Additionally, encouraging and supporting Black, Asian, and Latine people in civic engagement will help empower these groups to make environmental decisions for themselves and their communities.

I am looking for interview participants who:

- Spend time in nature,
- Are 18-25,
- And are Black, Asian, or Latinx.

All participants will be eligible for a drawing for a $50 digital gift card, with a 1 in 6 odds of being selected. If you're interested in joining me for a 30-45 minute virtual interview as part of this research, please click [here](https://virginiatech.qualtrics.com/jfe/form/SV_8hKwBmBOEGdhrQa)to learn more and answer some initial screening questions, or email me at aidabagheri@vt.edu.

Thanks so much for your help,

Aida Bagheri Hamaneh

Graduate Research Assistant

Department of Fish and Wildlife Conservation

Virginia Tech | Latham 340

*Sample Social Media Post*

Hey There! Want to contribute to a study on supporting diversity in outdoor spaces? I am a master’s student at [removed for anonymized review] conducting interviews develop a greater understanding of how to support BIPOC in outdoor spaces (IRB 22-113).). I am looking for interview participants from all over the US who:

- Participate in outdoor recreation
- Are 18-25
- And are Black, Asian, or Latinx.

All participants will be eligible for a drawing for a $50 digital gift card, with a 1 in 6 odds of being selected.

If you're interested in joining me for a 30-45 minute virtual interview as part of this research, please click [here](https://virginiatech.qualtrics.com/jfe/form/SV_8hKwBmBOEGdhrQa?fbclid=IwAR0Fr7o1k-JcebdTpSNIeyLJDHmuRybxjJr0Lm75WQSusCXGtQ_9t2aZUnE) to learn more and answer some initial screening questions, or email me at aidabagheri@vt.edu.


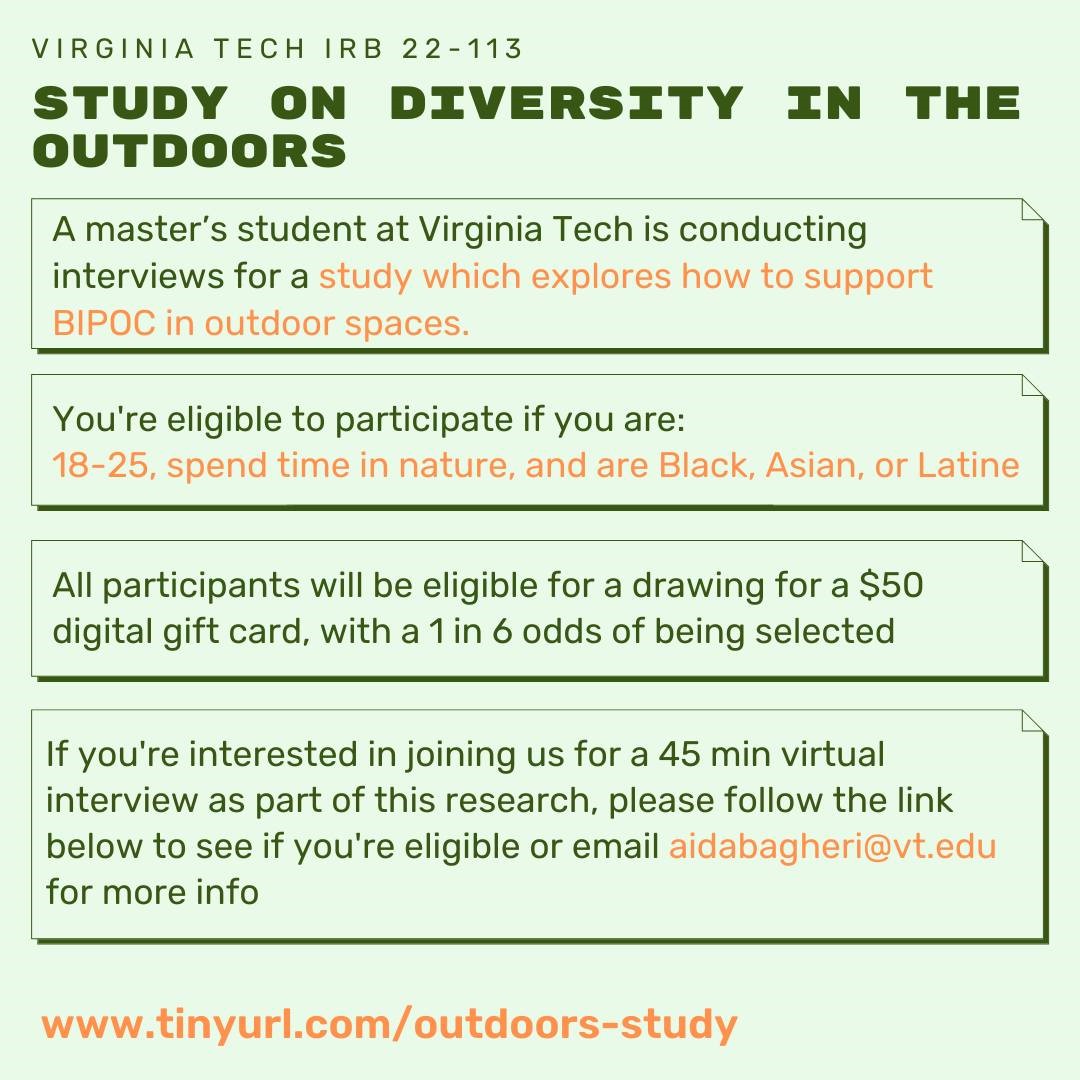


*Selection Email*

Email subject: [removed for anonymized review] in the Outdoors Virtual Interview: You have been selected

Dear [participant name]

You have been selected to participate in a virtual interview with [removed for anonymized review] about your experiences in the outdoors and with environmental civic engagement (IRB # IRB 22-113).

By sharing your thoughts and experiences, you can help us better understand what factors support Black, Asian, and Latinx folks in the outdoors and in environmental civic engagement. During the meeting, we’ll ask you a series of questions about your experiences. The interview should take approximately 30-45 minutes, and it will be audio-recorded to allow us to accurately capture your thoughts. Your responses will not be associated with your name. There are no known risks associated with participating in the study, and your participation is completely voluntary. Results from this research study will be used for peer-reviewed publications, a master’s thesis, presentations, and reports for participants.

To sign up to participate in this study, please use the following link to schedule a time to participate: <https://calendly.com/dei-study/interview>.

For more information on the study, please review the attached information sheet. If you have any questions regarding this study or would like more information before signing up for the interview, please reply to this email or email aidabagheri@vt.edu.

I look forward to talking with you!

Sincerely,

Aida Bagheri Hamaneh

Graduate Research Assistant

Department of Fish and Wildlife Conservation

Virginia Tech | Latham 340

*Reminder Email*

Email subject: We’d Like to Interview you About your Experiences in the Outdoors

Dear [participant name],

We’re interested in interviewing you as part of a [removed for anonymized review] on your experiences in the outdoors and with environmental civic engagement. By sharing your thoughts and experiences, you can help us better understand what factors support Black, Asian, and Latinx folks in the outdoors and in environmental civic engagement. During the meeting, we’ll ask you a series of questions about your experiences.

The interview should take approximately 30-45 minutes, and it will be audio-recorded to allow us to accurately capture your thoughts. Your responses will not be associated with your name. There are no known risks associated with participating in the study, and your participation is completely voluntary. Results from this research study will be used for peer-reviewed publications, a master’s thesis, presentations, and reports for participants.

To sign up to participate in this study, please use the following link to schedule a time to participate: <https://calendly.com/dei-study/interview>.

For more information on the study, please review the attached information sheet. If you have any questions regarding this study or would like more information before signing up for the interview, please reply to this email or email [removed for anonymized review].

I look forward to talking with you!

Sincerely,

Aida Bagheri Hamaneh

Graduate Research Assistant

Department of Fish and Wildlife Conservation

Virginia Tech | Latham 340

*Confirmation Email:*

Email subject: Confirming Interview for Study on Diversity in the Outdoors

Dear [Participant Name],

Thank you for your interest in this research study. This email is to confirm our scheduled interview for (time) on (date). Below is the zoom link that you may use for the interview. If you do not have internet access that allows for web-conferencing, you can call this number instead (zoom number and code).

I’m happy to answer any questions that you may have. I’m looking forward to speaking with you and if you have any questions please contact me at [aidabagheri@vt.edu](mailto:aidabagheri@vt.edu).

Sincerely,

Aida Bagheri Hamaneh

Graduate Research Assistant

Department of Fish and Wildlife Conservation

Virginia Tech | Latham 340

*Thank you email*

Email subject: Thank You for Your Participation in Our Study on Diversity in the Outdoors

Dear [Participant Name],

Thank you for taking the time to speak with me today. I appreciated hearing about your experiences with outdoor recreation and in environmental civic engagement. Once we have finalized the findings from this study, we will email you with a link to a summarized report of our results. You can expect a link to the report within the next 6-9 months.

Thank you again for your time.

Best,

Aida Bagheri Hamaneh

Graduate Research Assistant

Department of Fish and Wildlife Conservation

Virginia Tech | Latham 340
