# Supplementary material for: Investigating pathways to environmental civic engagement for diverse communities
Source: Environ Manage. 2026 Jan 7;76(2):61. doi: 10.1007/s00267-025-02356-2 (PMC12779674; doi:10.1007/s00267-025-02356-2)
Supplement: Supplementary file 2 — Appendix 2 [file 267_2025_2356_MOESM2_ESM.docx]

Appendix 2

*Organizations Contacted for Recruitment*

Alabama Black Belt Adventures

Amherst College -Outing Club

Basecamp: outdoor jobs and more

BIPOC Mountain Collective

BIPOC Outdoors Twin Cities

Black Adventure Crew

Black folks camp too

Black Girls Hike RVA

Black Girls Surf

Black Girls Trekkin

Black Hunters and Fishing

Black Outside Inc.

Brown Folks Fishing

Brown People Camping

Center for diversity in the environment

Climbers of color

Colorado Mountain Club

Community Nature Connection

Diversify whitewater

Georgia State University - Dunwoody Outdoor Adventure Club

Georgia State University - Student Environmental Team

Get Out Stay Out

Great Outdoors LA

HECHO (Hispanics enjoying camping, hunting, and the outdoors)

Hunters of Color

In Solidarity Project

Interim CDA

Justice Outside

Latino Outdoor DMV

Latino Outdoors

National Brotherhood of Skiers

Nature for All

Nuestra Tierra

Outdoor Alliance

Outdoor Asian

Outdoor Industry Association

Outdoors Empowered Network

PDX People of Color Outdoors

PGM One

Pomona College -Black Student Union

Pomona College's Latinx Alliance

Queer Nature

Rutgers Outdoors Club

Rutgers Wildlife Society

Sierra Club - North Star Chapter

Soul Trak

The Brown Ascenders

Trail Mixed

UC Riverside - CalPIRG

UCLA - Backpacking club

UCLA - Bruin Birding Club

UCLA - Environmentalists of Color Collective

University of Houston Horticultural Society

University of Houston Wall Crawlers

University of Nevada, Las Vegas - Audubon Student Conservation Chapter

University of Nevada, Las Vegas - Ecology, Conservation, and Evolution Club

University of Nevada, Las Vegas - Mountain Club

University of Nevada, Las Vegas - Student Sustainability Council

Venture Out Project

Venture Outdoors

Vibe Tribe Adventures

Virginia Tech - Bird Club

Virginia Tech - Black Students in STEM

Virginia Tech - Climbing Club

Virginia Tech - Horticulture Club

Virginia Tech - LAIGSA

Virginia Tech - MANRRS

Virginia Tech - Venture Out

Virginia Tech - Wildlife Society

Virginia Tech -Art for Environmental Justice

Virigina Tech - Outdoor Club

We Got Next

Wild Diversity

Women Who Hike
