# Supplementary material for: Investigating pathways to environmental civic engagement for diverse communities
Source: Environ Manage. 2026 Jan 7;76(2):61. doi: 10.1007/s00267-025-02356-2 (PMC12779674; doi:10.1007/s00267-025-02356-2)
Supplement: Supplementary file 3 — Appendix 3 [file 267_2025_2356_MOESM3_ESM.docx]

**Appendix 3**

**Supplemental Information on Methods**

Interview sampling and data collection

Participating groups circulated our recruitment materials, including a Qualtrics screening survey (Appendix 4). The survey filtered potential participants based on their age (i.e., 18-25), ethno-racial background (i.e., Black, Asian, or Latine heritage), and participation in nature-based activities (8+ outings a year). Although participants were not first screened out for participation in environmental civic engagement, only interviewees who indicated they participated were included in this study. We supplemented these recruitment efforts with snowball sampling, where we relied on our interviewees to share our recruitment materials with relevant members of their networks.

For individuals who completed the screening survey and met the requirements, we emailed further details and a link to schedule an interview. We followed up with two reminder emails (Dillman et al. 2014). We contacted 131 eligible candidates for the interviews.

The Zoom interview was recorded (audio only) and transcribed using Zoom transcription software and later revised for accuracy on inqScribe. By using open-ended questions, we were able to investigate both the significance of previously identified factors with the CCW framework and explore themes that emerged from participants' descriptions. We tested preliminary questions on six individuals outside our sample who fit the criteria. We adjusted the interview script based on the outcomes of these tests before administering them to our sample.

We entered each interview participant into a drawing for one of ten $50 gift cards. Further details of our interview sampling approach are included in Appendices 3 and 4. Anonymized demographic data for all participants is included in Appendix 5.

Survey Sampling

Using quotas to match the target population can be an effective way to reduce sampling bias (Wardropper et al. 2021). We used several quotas, including race, gender, age, income, and U.S. region residency by aligning with distributions found in U.S. census data (US Census Bureau 2019, 2020, 2023). We integrated multiple quality and attention checks (Appendix 8). Our final sample only included responses that either failed fewer than four of our quality and attention checks or underwent individual review and were deemed reliable (Wardropper et al. 2021).

Survey data collection

*Measures of Community Cultural Wealth*

Scales were reviewed by [omitted for blinded review] social scientists and piloted by a class of undergraduate students. The results from the pilot survey were used to conduct an exploratory factor analysis (EFA) to test the unidimensionality of each scale. We performed the analysis using the *psych* package in R, with a principal axis factoring extraction method and Varimax rotation (Ravelle 2023). We removed scale items with a coefficient of .04 or lower and several other items according to feedback from the pilot test. These modifications led to the final versions of the scales.

Survey data analysis

*Exploratory Factor Analysis (EFA) of Community Cultural Wealth*

The EFA employed a principal axis factoring extraction method and an oblique rotation (promax). As for the number of factors to extract, we looked at parallel analyses and scree plots. We removed any items with factor loadings of ≤ 0.04 and ran the EFA again until all factor items were acceptable.

References

Dillman DA, Smyth JD, Christian LM (2014) Internet, phone, mail, and mixed-mode surveys: The tailored design method, 4th edn. Wiley, Hoboken, NJ.

Revelle W (2023) psych: Procedures for Psychological, Psychometric, and Personality Research. Northwestern University, Evanston, IL. R package version 2.5.6. Available at: https://CRAN.R-project.org/package=psych.

U.S. Census Bureau (2019) American Community Survey (ACS) 1-year estimates. Available at: https://www.census.gov/newsroom/press-kits/2020/acs-1year.html.

U.S. Census Bureau (2020) 2020 ACS 1-year experimental data release. Available at: https://www.census.gov/programs-surveys/acs/data/experimental-data.html.

U.S. Census Bureau (2023) American Community Survey (ACS) — program page and 2023 data release overview. Available at: https://www.census.gov/programs-surveys/acs.html.

Wardropper CB, Dayer AA, Goebel MS, Martin VY (2021) Conducting conservation social science surveys online. Conservation Biology 35:1650–1658. https://doi.org/10.1111/cobi.13747.
