# Supplementary material for: Investigating pathways to environmental civic engagement for diverse communities
Source: Environ Manage. 2026 Jan 7;76(2):61. doi: 10.1007/s00267-025-02356-2 (PMC12779674; doi:10.1007/s00267-025-02356-2)

## **Appendix 4**

*Interview Screening Survey*

The questionnaire below only includes relevant sections used in this paper. The complete questionnaire is part of a larger study and can be available upon request


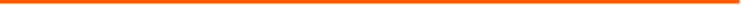


# **Consent**

Virginia Tech is conducting a national study to learn more about factors that initially drive and continually support Black, Asian, and Latinx Americans with their interactions with nature and participation in environmental civic engagement (IRB Protocol 22-113). As a component of this study, we will be conducting 30-45 minute virtual interviews with participants from all over the US who interact with nature, are 18-25, and are Black, Asian, or Latinx. By sharing your thoughts and experiences in these interviews, you can help us gain a better understanding of how to support all Black, Asian, and Latinx people in outdoor spaces.

Your participation in this study is completely voluntary and you can stop participating in the research study at any time, for any reason, and it will not be held against you. There are no right or wrong answers to the screening survey or the subsequent interview questions. Your responses will never be presented in a way that could be connected to your identity. The results of this study will be published as a graduate thesis, presentations, reports, and journal articles. There are no known risks or benefits to participating in this research study.

If you have questions regarding this study or would like more information, please contact Aida Bagheri Hamaneh at aidabagheri@vt.edu. If you have questions or concerns about this study's conduct or about your rights as a research subject, you may contact the Virginia Tech IRB at 540-231-3732 or irb@vt.edu.

Do you consent to participate in this research study?


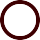
Yes, I agree to participate in this research study


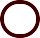
No, I do not agree to participate in this research study

# **Demographics**

What is your ZIP code for most of the year?


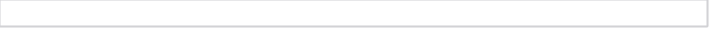


How old are you?


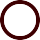
Under 18


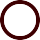
18-25


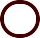
26-30


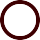
31-35


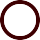
36-45


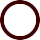
46-65


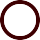
66+

What is your gender?


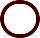
Male


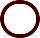
Female


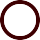
Non-binary / third gender


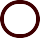
Prefer not to say


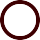
Prefer to self-describe
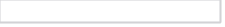


Are you of Hispanic, Latino, or Spanish Origin?


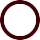
Yes


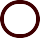
No

How would you describe yourself? (Please select all that apply.)


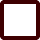
American Indian or Alaska Native


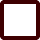
Asian


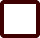
Black or African American


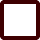
Native Hawaiian or other Pacific Islander


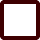
White

What is the highest degree or level of school you have completed? (If currently enrolled, highest degree received.)


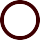
No schooling completed


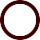
Some high school, no diploma


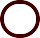
High school graduate (diploma or equivalent)


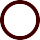
Some college, no degree


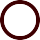
Associate's degree
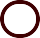
Bachelor's degree
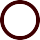
Graduate degree

How did you hear about this study?


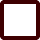
Outdoors Empowered


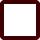
HECHO


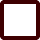
Latino Outdoors


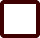
HBCUs Outside


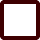
Next100 Coalition
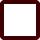
Black AF in STEM


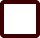
The Black Ecologists Section of the ESA


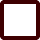
500 Women Scientists


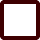
Scientist Sentinels


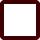
Other
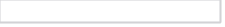


# **Interactions With Nature**

Next we are interested in your interactions with nature

In which of the following ways have you participated in outdoor recreation in the past 12 months?


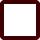
Trail-based activities (hiking, jogging, backpacking, off-roading etc.)


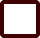
Water-based activities (kayaking, swimming, stand-up paddle boarding, etc.)


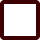
Winter outdoor activities (skiing, sledding, ice skating, etc.)


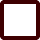
Wildlife-related activities (hunting, fishing, birdwatching, etc.)


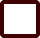
Outdoor sports (soccer, football, tennis, golf, etc.)


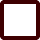
Camping (tent camping, RVs)


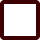
Picnicking


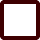
Other
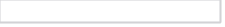


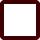
I do not participate in any of the activities.

In the past 12 months, about how many days did you spend engaging in the activities mentioned above?


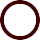
1-7


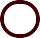
8-14


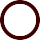
15-30


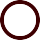
31-45


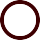
46+


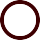
Never

# **Civic Engagement**

We are also interested in your civic engagement related to the environment

Civic engagement is the way that people are involved in the decision-making processes in their communities to improve conditions for themselves and others.

In the past 5 years, have you participated in any of the following forms of environmental civic engagement? (Please select all that apply.)


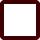
Signing petitions (written or email)


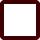
Volunteering for environmental advocacy groups


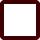
Protesting for environmental issues


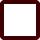
Donating money to support environmental causes


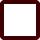
Boycotting for environmental causes


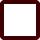
Voting to support environmental causes


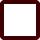
Writing letters in support of environmental causes


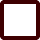
Other
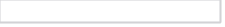


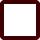
I do not participate in any of the activities.

# **Name and Email**

Thank you for telling us about yourself and your experiences. Finally, we need to know how to get in touch with you if you are selected to participate

Please provide your name and email address

Name
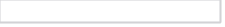


Email address
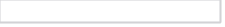

Supplement: Supplementary file 4 — Appendix 4 [file 267_2025_2356_MOESM4_ESM.docx]
