# Supplementary material for: Investigating pathways to environmental civic engagement for diverse communities
Source: Environ Manage. 2026 Jan 7;76(2):61. doi: 10.1007/s00267-025-02356-2 (PMC12779674; doi:10.1007/s00267-025-02356-2)
Supplement: Supplementary file 5 — Appendix 5 [file 267_2025_2356_MOESM5_ESM.docx]

APPENDIX 6

*Anonymized Demographic Data for Interview Participants*

| ID | Gender Identity | Latine | Race | Education |
| --- | --- | --- | --- | --- |
| 1 | Non-binary | Yes | White | Bachelor's degree |
| 2 | Non-binary | Yes | White | Bachelor's degree |
| 3 | Female | Yes | White | Bachelor's degree |
| 4 | Female | No | Black or African American | Bachelor's degree |
| 5 | Male | No | Black or African American | Bachelor's degree |
| 6 | Female | No | Asian | Some college, no degree |
| 7 | Female | No | Asian | Some college, no degree |
| 8 | Female | No | Asian | Bachelor's degree |
| 9 | Female | Yes | Asian | Associate's degree |
| 10 | Female | No | Black or African American | Bachelor's degree |
| 11 | Male | Yes | White | Some college, no degree |
| 12 | Female | No | Asian | Associate's degree |
| 13 | Male | Yes | White | Bachelor's degree |
| 14 | Female | Yes | Black or African American | Graduate degree |
| 15 | Female | No | Black or African American | Some college, no degree |
| 16 | Male | Yes | White | Bachelor's degree |
| 17 | Non-binary | No | Asian | Some college, no degree |
| 18 | Female | Yes | Asian | High school graduate (diploma or equivalent) |
| 19 | Female | No | Black or African American, White | Bachelor's degree |
| 20 | Non-binary | No | Asian | Bachelor's degree |
| 21 | Female | No | Black or African American, White | Bachelor's degree |
| 22 | Male | Yes | American Indian or Alaska Native, Black or African American | Some college, no degree |
| 23 | Female | Yes | Black or African American | Graduate degree |
| 24 | Male | Yes | White | Bachelor's degree |
| 25 | Female | No | Asian | Bachelor's degree |
| 26 | Female | Yes | White | High school graduate (diploma or equivalent) |
| 27 | Female | Yes | White | Bachelor's degree |
| 28 | Female | No | Asian | Bachelor's degree |
| 29 | Female | Yes | American Indian or Alaska Native, Asian, White | Bachelor's degree |
| 30 | Female | No | Asian, White | Graduate degree |
| 31 | Female | No | Asian | Graduate degree |
