# Supplementary material for: Investigating pathways to environmental civic engagement for diverse communities
Source: Environ Manage. 2026 Jan 7;76(2):61. doi: 10.1007/s00267-025-02356-2 (PMC12779674; doi:10.1007/s00267-025-02356-2)
Supplement: Supplementary file 6 — Appendix 6 [file 267_2025_2356_MOESM6_ESM.docx]

**Appendix 5**

*Interview scripts*

*Hello and thank you for taking the time to talk with me today. My name is Aida Bagheri*

*Hamaneh and I am a graduate student at Virginia Tech.*

*As a part of my thesis, I am conducting a national study to learn more about what supports Black, Asian, and Latinx people in outdoor recreation and civic engagement. In this interview, I will ask you some questions about your experiences with activities in natural settings, civic engagement, and environmental education. The interview should be about 30-45 minutes long. By participating in these interviews, you will help us gain a better understanding of how to support all Black, Asian, and Latinx people in outdoor spaces.*

*Please keep in mind that there are no right or wrong answers and that your responses will be completely confidential and never associated with your name, so please feel free to speak your mind openly. Your participation in this interview is also completely voluntary, and you can choose to skip questions or leave the conversation at any time. We will be audio recording this meeting to ensure that we accurately capture your responses.*

*Thank you again for your participation, do you have any questions before we begin?*

*Let's start by talking about nature and the ways that you interact with nature*

- What comes to mind when you think of nature? (Taylor)

1. **Interactions with Nature**
   1. What activities do you enjoy doing in natural settings or in the outdoors?
      1. Can you describe these activities? (Do you view wildlife, go boating, or fishing?)
   2. How did you first start [reference activities they mentioned]?
      1. Were there any significant events which you believe contributed to your interest in these activities? (Peters Grant)
         1. How about any significant people?
         2. How was this event/person significant?
   3. Since then, what has supported your continued engagement with these activities?
2. **Environmental Education/Interaction with Nature**

*Thank you for telling me about your experiences with nature. Now I hope to learn more about any environmental education experiences you might have had in your life. That would be any program or place where you learned more about the environment, and developed skills and understanding about how to solve the world’s environmental problems (NAAEE).*

- 1. Have you ever participated in an environmental education program?
     1. For example, have you ever experienced environmental education during a school field trip, at a nature center, zoo, or botanical garden?
     2. If so, can you describe these experiences?
     3. How did these experiences impact you?
  2. Have these experiences encouraged your *initial* interest in [reference activities they mentioned]?
     1. If so, in what ways?
  3. Have these experiences supported your continued engagement in [reference activities they mentioned]? (Such as giving skills or information on what to do or where to go, or introducing you to organizations or people?)
     1. If so, in what ways?

1. **Cultural Capital/Interaction with Nature**

*Thank you, now I’m going to ask you more questions about your engagement in [reference activities they mentioned].*

- 1. How have your parents responded to your interest in [reference activities they mentioned]? (Gonzalez)
     1. Have they encouraged your participation in these activities? How?
     2. For example, have they offered to drive you places, or pay for things, or given you skills or information on what to or where to go?
  2. Have members of your community outside of your family supported your engagement in these activities? (This can include organizations, friends, educators) (Gonzalez)
     1. In what ways?
     2. Are there any community resources that directly support your engagement in these activities (community groups, church groups, etc)? (Gonzalez)
     3. Do you generally engage in these activities alone or with others?
        1. Are these activities organized and planned by someone else (someone you know or an organization/person unfamiliar to you)
  3. What strategies, if any, do you use to maneuver through these activities? Such as getting information like finding where to go, or information about skills or equipment you might need. (Gonzalez)
     1. What strategies did you learn from your family and community that helped guide you through these activities? (Gonzalez)
  4. Were there any certain people, organizations, or groups that you felt you had to struggle against to participate in these activities? (Gonzalez)
     1. How did you persevere through that?(Gonzalez)

1. **Civic Engagement/Transformative Resistance**

*Thank you for sharing your thoughts and experiences. Now I would like to talk more about your participation in civic engagement related to the environment. By civic engagement I mean ways that you participate in your community or government in order to improve environmental conditions. Before I start asking questions about civic engagement, I’ll post a few examples in the chat so you can get an idea of what I mean. Please keep these examples in mind when answering questions, but remember that it's not a complete list so feel free to speak on other types of civic engagement as well. (In Zoom Chat: Community problem solving, Regular volunteering for an organization involved with environmental causes, Fundraising for an environmental cause, Donating to an environmental Cause, Regular voting to support environmental causes, Advocating for environmental issues, Campaign contributions with environmental causes in mind, Volunteering for candidate or political organizations because they support environmental causes, Contacting officials, Contacting media, Protesting, Signing petitions, Boycotting, Buycotting, Canvassing)*

- 1. In what ways, if any, do you participate in civic engagement related to the environment?
     1. Such as (but not limited to) voting, activism, advocating, participating in public hearings, making public comments (Alder et al.)
     2. Could you describe?
  2. What first encouraged you to participate in civic engagement related to the environment?
     1. Were there any significant events which you believe contributed to your involvement in civic engagement related to the environment?(Peters Grant)
        1. How about any significant people?
        2. How was this event/person significant?
     2. Can you describe your community’s (or neighborhood’s or friends) involvement in environmental issues?
        1. Are environmental issues a big concern in your community?
        2. Does this affect your participation in civic engagement? How?
     3. Earlier we talked about your experiences with environmental education, do you feel like those experiences influence your civic engagement? If so, how?
  3. What supports your continued participation in civic engagement related to the environment?
     1. Does your family or community support your participation in civic engagement related to the environment?
  4. What strategies, if any, do you use to maneuver through civic engagement processes?
     1. What strategies did you learn from your family and community that helped you navigate civic engagement processes?
  5. Were there any certain people, organizations, or groups that you felt you had to struggle against to participate in these activities? (Gonzalez)
     1. How did you persevere through that?(Gonzalez)

*Before we end, is there anything you would like to share with me that I didn’t cover in the interview?*

*Thank you for your participation! The results of these interviews will be published as a report, graduate thesis, and journal articles. If you’re interested, I can send you a short report of the results via email. Please reach out to me via phone or email if you have other questions or comments.*
