# Supplementary material for: Investigating pathways to environmental civic engagement for diverse communities
Source: Environ Manage. 2026 Jan 7;76(2):61. doi: 10.1007/s00267-025-02356-2 (PMC12779674; doi:10.1007/s00267-025-02356-2)
Supplement: Supplementary file 7 — Appendix 7 [file 267_2025_2356_MOESM7_ESM.docx]

**APPENDIX 7**

**Code Book**

| **Code** | **Description** | **Example** | **# of participants who mentioned** |
| --- | --- | --- | --- |
| Connection to nature | Describing an emotional, cognitive, or spiritual connection to nature | “I think being in really amazing landscapes is just really inspiring, and it’s very spiritual for me in a way. I didn’t grow up super religious, but I think I feel most connected with things outside of me when I am just out in nature | 13 |
| Continued participation | People, actions, or events that supported the participants continued participation or interest | “I think it's that the environmental jobs that I've worked in have had affinity spaces related to gender and race that have helped me feel more comfortable being in environmental spaces.” | 31 |
| Environmental education  As a child  As an adult | A program or place where individuals learn more about the environment, and develop skills and understanding about how to address global challenges (North American Association for Environmental Education) | “I can trace it back to learning about global warming and things like that in school, and just understanding the importance of the damage that we cause to the planet.” | 30 |
| Environmental justice knowledge | Knowledge of the unequal distribution of the benefits and burdens of the environment | “We didn’t have access to any parks that we could really go to...and we had some of the worst water quality in the state of Texas, some of the worst air pollution... I started realizing how my own community was really impacted and disadvantaged by all these factors” | 5 |
| Experiences of environmental degradation | Personal experiences of negative environmental impacts | “I come from a coastal community, and it was really big also seeing the rising sea levels. They took out a good section of a neighborhood nearby. And these people no longer had a place to live...” | 17 |
| Familial capital  Instrumental support  Emotional support  Community  Cultural knowledge | The cultural knowledge and support gained from family and community (Yosso, 2005) | “Most of it has just been knowledge that my family has or that I’ve learned from my Community...” | 23 |
| Initial interest/Initial participation | People, actions, or events that supported the participants initial participation or initial interest | “My AP environmental science teacher. She was definitely a hippie. And kind of drilled it into our heads like, if you don’t engage, they’re not gonna change anything. So, that was like a ‘oh, okay, gotta start doing stuff to help’.” | 31 |
| Knowledge of environmental issues | Exhibits knowledge of environmental issues (e.g. biodiversity loss, climate change, waste-disposal, ocean acidification, etc.) without stating a clear source of that knowledge | “Water pollution is actually really, really bad. Like it's crazy that there's a whole island of plastic somewhere floating in the ocean.” | 14 |
| Navigational capital | Skills or knowledge needed to maneuver through institutions made without Black, Indigenous, and people of color in mind (Yosso, 2005) | “When I was in college, there was the outdoor club, and they had all the gear for you and there was basically a guide that could help you do all the things.” | 29 |
| Resistant capital | An understanding of the structures of inequality and the motivation, skills and knowledge used to challenge that inequality (Yosso, 2005) | “I think just seeing and advocating for change and that - that more people can be involved, especially minorities. It’s important me to try to do my part to advocate for the change, and that more people can be involved, but the hope that everyone has equal access to their civic duties, and being able to partake in them.” | 16 |
| Self-efficacy  Political efficacy | A person’s belief in their capacity to execute a specific behavior | “... So I think that gave me confidence and interest to be like ‘yeah, I can learn how to do this’” | 11 |
| Social capital  Organization/club  Affinity group  Educator  Mentor  Friends  Fellow marginalized individual  School or school organization  Social media | Connections with and support from people and community resources (Yosso, 2005) | “I like to reach out to someone who I trust and has personal experience. Because I think that they may be able to give me some more advice of what they wish they knew before they started, that they think would be pertinent to me because they know me” | 29 |
| Supporting future generations  Shared experiences | Expressing a desire to contribute to the well-being and support of upcoming generations, whether referring to the younger generation or one's own children. | “I don’t have kids yet, but like I want to have kids, and wanting to leave a better environment for them, and wanting for them to have a relationship with the environment around them” | 7 |
