# Supplementary material for: Investigating pathways to environmental civic engagement for diverse communities
Source: Environ Manage. 2026 Jan 7;76(2):61. doi: 10.1007/s00267-025-02356-2 (PMC12779674; doi:10.1007/s00267-025-02356-2)
Supplement: Supplementary file 8 — Appendix 8 [file 267_2025_2356_MOESM8_ESM.docx]

Appendix 8

*Data Quality*

We included several quality, attention, and time checks in the survey. Three of these questions were straight lining questions where respondents were flagged if their responses to a block of questions which included an inverse worded question had a standard deviation of zero. We included two logic checks. For example, if a respondent indicated that they did participate in nature-based activities earlier in the survey and then claimed that they did not later on in the survey, their entry was flagged. For the time check, we set the minimum completion time at ⅕ the median of all responses, and flagged all responses completed faster than the minimum time. An attention check question prompted respondents to choose a picture of a “natural area” after defining the concept. Incorrect responses were flagged. Lastly, when asking about environmental education experiences, we included a phony environmental education program as a trap question. All respondents with four or more flags were removed from the sample. Remaining respondents who had one, two, or three flags were all individually checked for reliability. Those considered unreliable were removed from the final sample.
