# Supplementary material for: Investigating pathways to environmental civic engagement for diverse communities
Source: Environ Manage. 2026 Jan 7;76(2):61. doi: 10.1007/s00267-025-02356-2 (PMC12779674; doi:10.1007/s00267-025-02356-2)
Supplement: Supplementary file 9 — Appendix 9 [file 267_2025_2356_MOESM9_ESM.docx]

### Appendix 9

*Survey Instrument*

### ✱ Thank you for participating in our survey!

We are conducting a national study **to learn more about what supports People of Color who live in the US with their participation in nature-based activities and environmental civic engagement** (IRB Protocol #22-113). By sharing your thoughts and experiences in this survey, you can help us gain a better understanding of how to support people's diverse experiences in outdoor spaces.

This survey should take about 15-20 minutes to complete. Your participation in this study is **completely voluntary and anonymous**. You can stop participating in the research study at any time, for any reason, and it will not be held against you. There are no right or wrong answers to the survey questions. Your responses will never be presented in a way that could be connected to your identity. The anonymous results of this study will be published as a graduate thesis, presentations, reports, and journal articles. There are no known risks or benefits to participating in this research study.

If you have questions regarding this study or would like more information, please contact Aida

Bagheri Hamaneh at aidabagheri@vt.edu. If you have questions or concerns about this study's

conduct or about your rights as a research subject, you may contact the Virginia Tech IRB at 540-

231-3732 or irb@vt.edu.

### Do you consent to participate in this research study?


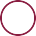
 Yes, I agree to participate in this research study


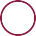
 No, I do not agree to participate in this research study

# To begin, we have a few questions about you

✱ How many days per year do you participate in **nature-based activities**?

Note: Please consider “nature-based activities" to include **spending time in natural areas** such as the woods, local parks, a lake or beach, or other features of the natural environment.


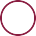
 Never


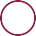
 1 to 11 days per year


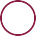
 12 to 51 days per year
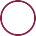
 Over 52 days per year

✱ **Civic engagement related to the environment** is the way that you **participate in your community or government** to **create positive environmental change** (this can include voting,

petitioning, volunteering, donating, and advocating for the environment).

Thinking about the past **5 years**, have you participated in civic engagement related to the environment?


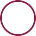
 No, I have not participated


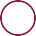
 Yes, I participated sometimes


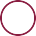
 Yes, I participated rarely


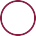
 Yes, I participated often

✱ In what year were you born?

*(Please select your birth year from the drop-down list.)*


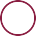
 After 2004


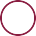
 2004


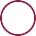
 2003


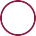
 2002


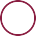
 2001


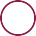
 2000


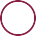
 1999


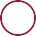
 1998


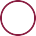
 1997


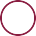
 1996


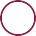
 1995


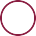
 1994


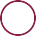
 1993


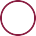
 1992


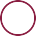
 1991


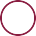
 1990


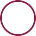
 1989


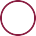
 1988


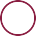
 1987


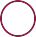
 1986


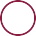
 1985


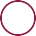
 1984


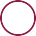
 1983


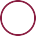
 1982


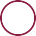
 1981


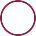
 1980


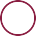
 1979


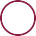
 1978


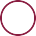
 1977


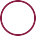
 1976


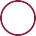
 1975


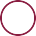
 1974


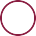
 1973


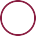
 1972


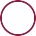
 1971


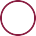
 1970


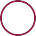
 1969


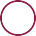
 1968


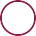
 1967


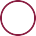
 1966


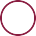
 1965


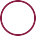
 1964


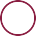
 1963


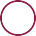
 1962


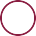
 1961


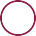
 1960


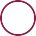
 1959


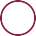
 1958


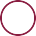
 1957


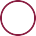
 1956


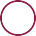
 1955


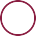
 1954


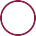
 1953


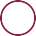
 1952


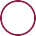
 1951


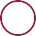
 1950


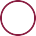
 1949


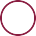
 1948


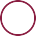
 1947


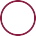
 1946


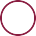
 1945


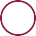
 1944


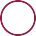
 1943


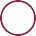
 1942


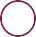
 1941


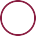
 1940


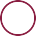
 1939


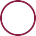
 1938


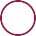
 1937


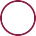
 1936


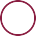
 1935


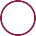
 1934


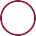
 1933


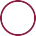
 1932


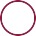
 1931


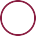
 1930


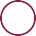
 1929


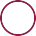
 1928


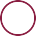
 1927


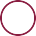
 1926


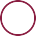
 1925


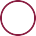
 1924


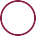
 1923


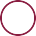
 1922


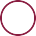
 1921

✱ Are you over 18?


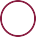
 Yes, I am 18 years or older.
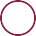
 No, I am under 18 years old.

✱ What is your racial or ethnic identity?

*(Please select all that apply.)*


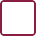
 African American/Black
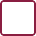
 East Asian


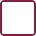
 Hispanic/Latinx Middle Eastern

American Indian/Alaskan Native Pacific Islander

South Asian

Southeast Asian White

Other (please specify)

✱ What region of the country do you live in?

**Northeast**: Connecticut, Maine, Massachusetts, New Hampshire, New Jersey, New York, Pennsylvania, Rhode Island, or Vermont

**South**: Alabama, Arkansas, Delaware, D.C., Florida, Georgia, Kentucky, Louisiana, Maryland, Mississippi, North Carolina, Oklahoma, South Carolina, Tennessee, Texas, Virginia, or West Virginia

**Midwest**: Illinois, Indiana, Iowa, Kansas, Michigan, Minnesota, Missouri, Nebraska, North Dakota, Ohio, South Dakota, or Wisconsin

**West**: Alaska, Arizona, California, Colorado, Hawaii, Idaho, Montana, Nevada, New Mexico, Oregon, Utah, Washington, or Wyoming

None of the above

✱ What is your **9-digit ZIP code** for most of the year? Note: [**Click here**](https://tools.usps.com/zip-code-lookup.htm?byaddress) to look up your 9-digit ZIP code.

✱ What is the **population** size of the town/city where you now live?

Note: [**Click here**](https://datacommons.org/place) to look up the population size of your home town.

Up to 4,999 people 5,000 to 9,999 people

10,000 to 49,999 people

50,000 to 99,999 people

100,000 to 249,999 people

250,000 to 499.999 people 500,000 people or more

✱ What is your gender?

Woman Man

Non-Binary

Prefer not to disclose Prefer to self-describe

What is your gender?

✱ What was your total household income in the last 12 months?

Less than $5,000 $5,000 to $9,999 $10,000 to $14,999

$15,000 to $19,999 $20,000 to $24,999 $25,000 to $29,999 $30,000 to $34,999 $35,000 to $39,999 $40,000 to $44,999 $45,000 to $49,999 $50,000 to $54,999 $55,000 to $59,999 $60,000 to $64,999 $65,000 to $69,999 $70,000 to $74,999 $75,000 to $79,999 $80,000 to $84,999 $85,000 to $89,999 $90,000 to $94,999 $95,000 to $99,999 $100,000 to $124,999 $125,000 to $149,999 $150,000 to $174,999 $175,000 to $199,999 $200,000 to $249,999

$250,000 and above

✱ What is the highest level of school that you have completed?

3rd grade or less Grades 4 to 8

Completed some high school

High school graduate or equivalent

Other post-high school vocational training Completed some college, but no degree Associate degree

College degree (such as B.A., B.S.)

Completed some graduate, but no degree Master's degree

Doctorate degree

### ✱ Now we would like to ask you some questions about civic engagement related to the environment.

*Please note: Civic engagement related to the environment is the way that you participate in your community or government to create positive environmental change.*

Thinking about the **last 5 years**, have you spent time participating in any of the following?

Volunteering for an organization involved with environmental causes

Volunteering for a candidate or political organization that supports environmental causes

No, not in the past 5 years

Yes, but not in the last 12

months Yes, in the last 12 months

| Participating as an active member of a national or local environmental organization (such as the Sierra Club, the Audubon Society, etc.) |  | | |
| --- | --- | --- | --- |
| Fundraising money for environmental issues or organizations |  |  |  |
| Signing an online or written petition to support an environmental initiative |  |  |  |
| Protesting for an environmental cause |  |  |  |
|  |  | Yes, but not in the last 12 |  |
|  | No, not in the past 5 years | months | Yes, in the last 12 months |
| Donating money to an environmental organization or  cause |  |  |  |
| Donating money to a candidate or political party that supports  environmental causes |  |  |  |
| Contacting local or national representatives to ask for assistance or to express your opinion about environmental  causes |  |  |  |
| Contacting the media (newspapers, TV, radio) to express your opinions regarding  protecting the environment |  |  |  |
| Working informally with others in your community to solve a  local environmental problem |  |  |  |
| Canvassing (or going door to door) to persuade people to support environmental initiatives |  |  |  |
|  |  | Yes, but not in the last 12 |  |
|  | No, not in the past 5 years | months | Yes, in the last 12 months |
| Trying to persuade others to support candidates or policies that support environmental  causes |  |  |  |

Buying a product because it's environmentally friendly or because the profits go towards environmental causes

NOT buying a product because of the environmental conditions under which the product is made, or because you dislike the environmental conduct of the company that produces it

Other forms of civic engagement related to the environment not mentioned above

✱ In the last question, you selected "other". What other types of civic engagement related to the environment do you participate in?

✱ How often do you vote for local or national candidates who support environmental causes?

Never Rarely

Sometimes Often

Always

✱ The statements below are about **how you first started participating in civic engagement related to the environment.**

I first began participating in civic engagement related to the environment because of...

Do not agree Slightly agree Moderately agree Mostly agree Completely agree

... a friend

... an organization, group, or

club

... a role model

... a family member

### ✱ This next series of questions will be about civic engagement related to the environment.

*Please note: Civic engagement related to the environment is the way that you participate in your community or government to create positive environmental change.*

Please indicate the degree to which you agree with each statement below.

Do not agree Slightly agree Moderately agree Mostly agree Completely agree

I know where to learn about civic engagement

processes (such as how to vote or how to reach out to a representative).

I know where to find information about environmental issues and actions that I can take to help.

I know where to find information about opportunities to participate in civic engagement related to the environment.

People in my ethnic/racial/cultural community face discrimination when participating in civic engagement related to the environment.

I do not feel confident searching for information about civic engagement related to the environment.

✱ Please indicate the degree to which you agree with each statement below.

Do not agree Slightly agree Moderately agree Mostly agree Completely agree

I draw on connections

with friends to participate in civic engagement related to the environment.

I draw on connections

with organizations, groups, or

clubs to participate in civic engagement related to the

environment.

My friends and I talk to each other about our civic engagement related to the environment.

My friends encourage me to

participate in civic engagement related to the environment

✱ Please indicate the degree to which you agree with each statement below.

|  | Do not agree | Slightly agree | Moderately agree | Mostly agree | Completely agree |
| --- | --- | --- | --- | --- | --- |
| My family encourages me to |  |  |  |  |  |
| participate in civic engagement |  |  |  |  |  |
| related to the environment. |  |  |  |  |  |
| I have helped my family |  |  |  |  |  |
| participate in civic engagement |  |  |  |  |  |
| related to the environment. |  |  |  |  |  |
| My family listens to me talk |  |  |  |  |  |
| about my participation in civic |  |  |  |  |  |
| engagement related to the |  |  |  |  |  |
| environment. |  |  |  |  |  |

✱ This set of statements is also about participation in **civic engagement related to the environment.**

*Remember, civic engagement related to the environment is the way that you participate in your community or government to create positive environmental change.*

Please indicate the degree to which you agree with each statement below.

Do not agree Slightly agree Moderately agree Mostly agree Completely agree

Participating in civic engagement related to the environment will help combat stereotypes about people in my ethnic/racial/cultural community.

Participating in civic engagement related to the environment will pave the way for future generations of people in my ethnic/racial/cultural community to participate.

I want to inspire other people in my ethnic/racial/cultural community to participate in civic engagement related to the environment.

Even when presented with obstacles, I am able to continue

participating in civic

engagement related to the environment.

I have developed strategies to navigate difficult people and situations while participating in civic engagement related to the environment.

### ✱ Next, we want to know more about how you participate in nature-based activities.

*Note: Please consider “nature-based activities" to include* ***spending time in natural areas*** *such as the woods, local parks, a lake or beach, or other features of the natural environment.*

How often do you participate in nature-based activities?

Never

A few times per year Every month

Every week

✱ In a **typical week**, how many days do you spend participating in nature-based activities?

*(Please select one from the drop-down list.)*

1 day a week 2 days a week 3 days a week 4 days a week 5 days a week 6 days a week 7 days a week

I do not participate in nature-based activities

✱ In a **typical month**, how many days do you spend participating in nature-based activities?

*(Please select one from the drop-down list.)*

1 day a month 2 days a month 3 days a month 4 days a month 5 days a month 6 days a month 7 days a month 8 days a month 9 days a month

10 days a month 11 days a month 12 days a month 13 days a month 14 days a month 15 days a month 16 days a month 17 days a month 18 days a month 19 days a month 20 days a month 21 days a month 22 days a month 23 days a month 24 days a month 25 days a month 26 days a month 27 days a month 28 days a month 29 days a month 30 days a month 31 days a month

I do not participate in nature-based activities

✱ In a **typical year**, how many days do you spend participating in nature-based activities?

*(Please select one from the drop-down list.)*

1 day a year 2 days a year 3 days a year 4 days a year 5 days a year 6 days a year 7 days a year 8 days a year 9 days a year 10 days a year 11 days a year 12 days a year 13 days a year 14 days a year 15 days a year 16 days a year 17 days a year 18 days a year 19 days a year

20 days a year 21 days a year 22 days a year 23 days a year 24 days a year 25 days a year 26 days a year 27 days a year 28 days a year 29 days a year 30 days a year 31 days a year 32 days a year 33 days a year 34 days a year 35 days a year 36 days a year 37 days a year 38 days a year 39 days a year 40 days a year 41 days a year

42 days a year 43 days a year 44 days a year 45 days a year 46 days a year 47 days a year 48 days a year 49 days a year 50 days a year 51 days a year 52 days a year 53 days a year 54 days a year 55 days a year 56 days a year 57 days a year 58 days a year 59 days a year 60 days a year 61 days a year 62 days a year 63 days a year 64 days a year

65 days a year 66 days a year 67 days a year 68 days a year 69 days a year 70 days a year 71 days a year 72 days a year 73 days a year 74 days a year 75 days a year 76 days a year 77 days a year 78 days a year 79 days a year 80 days a year 81 days a year 82 days a year 83 days a year 84 days a year 85 days a year 86 days a year

87 days a year 88 days a year 89 days a year 90 days a year

More than 90 days a year

I do not participate in nature-based activities

✱ In a **typical outing**, about **how many hours** do you spend participating in **nature-based activities**?

Note: Please consider “nature-based activities" to include spending time in natural areas such as the woods, local parks, a lake or beach, or other features and of the natural environment.

*(Please select one from the drop-down list.)*

Less than 1 hour 1 hour

2 hours

3 hours

4 hours

5 hours

6 hours

7 hours

8 hours

9 hours

10 hours

11 hours

12 hours

More than 12 hours

✱ Looking at the images below, **please select the image of a "natural area".**

Remember, natural areas include the woods, local parks, a lake or beach, or other features of the natural environment.

Option 1 Option 2

Option 3 Option 4

# For this next section, we would like to know more about you.

✱ Please indicate the degree to which you agree with each statement below.

Do not agree Slightly agree Moderately agree Mostly agree Completely agree

There is at least one person I know whose advice I really trust.

I feel that there is no one with

whom I can share my most private worries and fears.

When I need suggestions for how to deal with a personal problem I know there is someone I can turn to.

If I had to go out of town for a few weeks, someone I know would look after my home (the plants, pets, yard, etc.)

There is no one I could call on if I needed to borrow a car for a few hours.

If I got stranded 10 miles out of

town, there is someone I could call to come get me.

✱ Please indicate the degree to which you agree with each statement below.

|  | Do not agree | Slightly agree | Moderately agree | Mostly agree | Completely agree |
| --- | --- | --- | --- | --- | --- |
| Family values are an important part of my cultural background. |  |  |  |  |  |
| I know about my family’s cultural heritage/history. |  |  |  |  |  |
| I have role models in my family. |  |  |  |  |  |
| Family is not important to me. |  |  |  |  |  |
| I maintain a connection to my extended family. |  |  |  |  |  |
| My family provides me with emotional support. |  |  |  |  |  |
| I maintain a connection to my home community and culture. |  |  |  |  |  |

I learn a lot of valuable

knowledge from my family members.

✱ Please indicate the degree to which you agree with each statement below.

Do not agree Slightly agree Moderately agree Mostly agree Completely agree

I have developed strategies to navigate difficult people and situations.

I have succeeded despite barriers to my success.

I am confident in my ability to get through struggles.

✱ Please indicate the degree to which you agree with each statement below.

|  | Do not agree | Slightly Agree | Moderately agree | Mostly agree | Completely agree |
| --- | --- | --- | --- | --- | --- |
| I believe there are injustices in my ethnic/racial/cultural community. |  |  |  |  |  |
| I believe I will be able to make a difference in society. |  |  |  |  |  |
| I want to create a more just or equitable society. |  |  |  |  |  |
| I believe racism is a major factor for issues in society. |  |  |  |  |  |
| I want to make a difference in my racial/ethnic/cultural community. |  |  |  |  |  |

# Lastly, we would like to know more about your past experiences with environmental education.

✱ In this survey, environmental education can be defined as a process that helps people **learn more** about the environment and **develop skills** and understanding about how to **solve environmental problems** (this can include going on a school fieldtrip to a nature center, taking an environmental science class, or watching a video about environmental issues, etc.).

Have you ever taken part in any of the following types of environmental education before?

*(Please select all that apply.)*

Environmental education during elementary school, including school field trips Environmental education during middle school, including school field trips

Environmental education during high school, including school field trips Environmental education during college

Environmental education during professional training programs that offer licensure or certificates of completion

Environmental education program run by parks, museums, nature centers, or somewhere similar Environmental education as part of a 4H or FFA (Future Farmers of America) program

Environmental education as part of Girl Scouts or Boy Scouts or similar program Environmental education as part of a Johnson's Young Environmentalists program Environmental education through informational plaques at museums or parks

Environmental education through web/social media content

Environmental education through environmental movies or TV shows Other environmental education activity not listed above

None of the above

Please describe the environmental education program you participated in.

✱ In the last question, you selected "**Other**". Thinking about that environmental education experience

- how often did you participate in something like this?

Never

Once

Once in a while Regularly

✱ Thinking about your experiences with environmental education in **elementary school** - how often did you participate in something like this?

Never Once

Once in a while Regularly

✱ Thinking about your experiences with environmental education in **middle school** - how often did you participate in something like this?

Never Once

Once in a while Regularly

✱ Thinking about your experiences with environmental education in **high school** - how often did you participate in something like this?

Never Once

Once in a while Regularly

✱ Thinking about your experiences with environmental education in **college** - how often did you participate in something like this?

Never Once

Once in a while Regularly

✱ Thinking about your experiences with environmental education in **professional training programs** - how often did you participate in something like this?

Never Once

Once in a while Regularly

✱ Thinking about your experiences with environmental education run by a **park, museum, nature center**, or somewhere similar - how often did you participate in something like this?

Never Once

Once in a while Regularly

✱ Thinking about your experiences with environmental education through **4H or FFA** - how often did you participate in something like this?

Never

Once

Once in a while Regularly

✱ Thinking about your experiences with environmental education as part of **Boy Scout's, Girl Scout's**, or a similar program - how often did you participate in something like this?

Never Once

Once in a while Regularly

✱ Thinking about your experiences with environmental education through **plaques at museums or parks** - how often did you interact with something like this?

Never Once

Once in a while Regularly

✱ Thinking about your experiences with environmental education through **web/social media content** - how often did you interact with something like this?

Never Once

Once in a while Regularly

✱ Thinking about your experiences with environmental education through **environmental movies or TV shows** - how often did you watch something like this?

Never

Once

Once in a while

Regularly
