# Supplementary material for: Investigating pathways to environmental civic engagement for diverse communities
Source: Environ Manage. 2026 Jan 7;76(2):61. doi: 10.1007/s00267-025-02356-2 (PMC12779674; doi:10.1007/s00267-025-02356-2)
Supplement: Supplementary file 10 — Appendix 10 [file 267_2025_2356_MOESM10_ESM.docx]

**Appendix 10**

*Loadings of Exploratory Factor Analyses for social, familial, navigational, and resistant capital from the Community Cultural Wealth framework*

Table S10.1. The factor loadings from an EFA using the principal axis factoring extraction method with an oblique rotation (promax) for items used to evaluate CCW. The four factor model accounted for 54% of the variance (Bagheri Hamaneh et al. *in press*).

| Item Code | Item Text | *F1* | *F2* | *F3* | *F4* |
| --- | --- | --- | --- | --- | --- |
| Social Capital  (Cronbach’s ⍺ = 0.77  Mean = 3.78  SD = 0.94) |  |  |  |  |  |
| Social 1 | There is at least one person I know whose advice I really trust. | 0.01 | 0.06 | 0.07 | **0.58** |
| Social 2 | When I need suggestions for how to deal with a personal problem, I know there is someone I can turn to. | 0.01 | 0 | -0.01 | **0.75** |
| Social 3 | If I had to go out of town for a few weeks, someone I know would look after my home (the plants, pets, yard etc.) | 0.11 | -0.02 | 0.03 | **0.59** |
| Social 4 | If I got stranded 10 miles out of town, there is someone I could call to come get me. | 0.1 | 0.05 | 0.02 | **0.53** |
| Navigational Capital  (Cronbach’s ⍺ = 0.81  Mean = 3.70  SD = .95) |  |  |  |  |  |
| Navigational 1 | I have developed strategies to navigate difficult people and situations. | -0.02 | 0.08 | **0.63** | 0.06 |
| Navigational 2 | I have succeeded despite barriers to my success. | 0.01 | -0.1 | **0.89** | -0.05 |
| Navigational 3 | I am confident in my ability to get through struggles. | -0.06 | -0.07 | **0.8** | 0.11 |
| Familial Capital  (Cronbach’s ⍺ = 0.89  Mean = 3.65  SD = .95) |  |  |  |  |  |
| Familial 1 | Family values are an important part of my cultural background. | **0.6** | 0.1 | 0.01 | 0.07 |
| Familial 2 | I know about my family’s cultural heritage/history. | **0.69** | 0.01 | 0.08 | -0.09 |
| Familial 3 | I have role models in my family. | **0.77** | -0.07 | -0.06 | 0.07 |
| Familial 4 | I maintain a connection to my extended family. | **0.72** | -0.01 | -0.04 | 0.03 |
| Familial 5 | My family provides me with emotional support. | **0.73** | -0.13 | -0.06 | 0.2 |
| Familial 6 | I maintain a connection to my home community and culture. | **0.71** | 0.05 | 0.1 | -0.09 |
| Familial 7 | I learn a lot of valuable knowledge from my family members. | **0.8** | -0.03 | -0.08 | 0.06 |
| Resistant Capital  (Cronbach’s ⍺ = 0.82  Mean = 3.74  SD = .98) |  |  |  |  |  |
| Resistant 1 | I believe there are injustices in my ethnic/racial/cultural community. | -0.08 | **0.86** | -0.11 | 0.05 |
| Resistant 2 | I want to create a more just or equitable society. | 0.08 | **0.63** | 0.14 | -0.02 |
| Resistant 3 | I believe racism is a major factor for issues in society. | -0.14 | **0.82** | -0.11 | 0.08 |
| Resistant 4 | I want to make a difference in my racial/ethnic/cultural community. | 0.25 | **0.57** | 0.13 | -0.13 |

**Reference**

Bagheri Hamaneh A, Dayer AA, Drape AT, Chaves WA (*in press*) Bridging the nature gap: Supporting ethno-racial diversity in outdoor spaces. *Journal of Outdoor Recreation and Tourism.*
