# Supplementary material for: Investigating pathways to environmental civic engagement for diverse communities
Source: Environ Manage. 2026 Jan 7;76(2):61. doi: 10.1007/s00267-025-02356-2 (PMC12779674; doi:10.1007/s00267-025-02356-2)
Supplement: Supplementary file 11 — Appendix 11 [file 267_2025_2356_MOESM11_ESM.docx]

## **APPENDIX 11**

*Survey Respondent Characteristics*

Table S11.1. The characteristics of our survey respondents (n=1808) after being weighted by ethno-racial identity.

| **CHARACTERISTICS** | **PERCENTAGE** |
| --- | --- |
| **Race** |  |
| Latine | 46% |
| Black | 30% |
| Asian | 14% |
| Multiracial | 10% |
| **Gender** |  |
| Woman | 54% |
| Man | 46% |
| **Income** |  |
| Low (up to 49,999) | 39% |
| Middle (50,000 - 149,999) | 53% |
| High (150,000 or above) | 8% |
| **Education** |  |
| High school graduate or equivalent | 23% |
| Some college - college degree | 61% |
| Some graduate schooling - doctorate | 16% |
| **Environmental Education** |  |
| Previous EE experience | 83% |
| No previous EE experience | 17% |
| **Participation in Nature-Based Activities** |  |
| Participate in a typical year | 85% |
| Do not participate in a typical year | 15% |
| **Participation in Environmental Civic Engagement** |  |
| Participate | 65% |
| Do not participate | 35% |
| 25 or Above | 87% |
| **Region** |  |
| Northeast | 17% |
| South | 42% |
| Midwest | 14% |
| West | 27% |
