# Supplementary material for: Investigating pathways to environmental civic engagement for diverse communities
Source: Environ Manage. 2026 Jan 7;76(2):61. doi: 10.1007/s00267-025-02356-2 (PMC12779674; doi:10.1007/s00267-025-02356-2)
Supplement: Supplementary file 12 — Appendix 12 [file 267_2025_2356_MOESM12_ESM.docx]

**Appendix** **12**

*Parameter estimates and Odds Ratio*

**Table S10.1. General Environmental Civic Engagement**. Parameter estimates, standard error, and odds ratios for the effects of community cultural wealth, environmental education and other variables on general participation in civic engagement related to the environment. Values between brackets correspond to 95% confidence intervals.

| **Variables** | **Estimate** | **Odds ratio** | **p-value** |
| --- | --- | --- | --- |
| Social Capital | 0.07 [-0.04, 0.18] | 1.07 [0.96, 1.20] | 0.23 |
| Navigational Capital | 0.17 [0.07, 0.28] | 1.19 [1.07, 1.32] | 0.002 |
| Resistant Capital | 0.28 [0.18, 0.38] | 1.32 [1.20, 1.46] | <0.0001 |
| Familial Capital | 0.20 [0.08, 0.32] | 1.22 [1.08, 1.37] | 0.001 |
| Black or African American | -0.45 [-0.77, -0.14] | 0.64 [0.46, 0.87] | 0.005 |
| Asian or Pacific Islander | -0.24 [-0.59, 0.11] | 0.79 [0.56, 1.11] | 0.18 |
| Latine or Hispanic | -0.18 [-0.48, 0.11] | 0.83 [0.62, 1.12] | 0.22 |
| Age (>25 years old) | -0.16 [-0.41, 0.09] | 0.85 [0.67, 1.09] | 0.21 |

**Table S10.2. Signing Petitions**. Parameter estimates, standard error, and odds ratios for the effects of community cultural wealth, environmental education and other variables on signing petitions. Values between brackets correspond to 95% confidence intervals.

| **Variables** | **Estimate** | **Odds ratio** | **p-value** |
| --- | --- | --- | --- |
| Social Capital | 0.12 [0.00, 0.25] | 1.13 [1.00, 1.28] | 0.05 |
| Navigational Capital | 0.07 [-0.05, 0.19] | 1.08 [0.96, 1.21] | 0.23 |
| Resistant Capital | 0.47 [0.36, 0.58] | 1.60 [1.43, 1.79] | <0.0001 |
| Familial Capital | 0.03 [-0.10, 0.16] | 1.03 [0.91, 1.17] | 0.66 |
| Black or African American | -0.64 [-0.97, -0.30] | 0.53 [0.38, 0.74] | 0.0002 |
| Asian or Pacific Islander | -0.13 [-0.51, 0.25] | 0.88 [0.60, 1.28] | 0.50 |
| Latine or Hispanic | 0.17 [-0.14, 0.49] | 1.19 [0.87, 1.63] | 0.28 |
| Age (>25 years old) | -0.34 [-0.61, -0.07] | 0.71 [0.54, 0.94] | 0.01 |

**Table S10.3. Donating**. Parameter estimates, standard error, and odds ratios for the effects of community cultural wealth, environmental education and other variables on donating to environmental organizations or causes. Values between brackets correspond to 95% confidence intervals.

| **Variables** | **Estimate** | **Odds ratio** | **p-value** |
| --- | --- | --- | --- |
| Social Capital | 0.07 [-0.06, 0.19] | 1.07 [0.95, 1.21] | 0.28 |
| Navigational Capital | 0.12 [0.00, 0.24] | 1.13 [1.00, 1.27] | 0.05 |
| Resistant Capital | 0.32 [0.21, 0.43] | 1.37 [1.23, 1.54] | <0.0001 |
| Familial Capital | 0.18 [0.05, 0.30] | 1.19 [1.05, 1.36] | 0.01 |
| Black or African American | -0.19 [-0.53, 0.17] | 0.83 [0.59, 1.18] | 0.30 |
| Asian or Pacific Islander | 0.38 [-0.01, 0.77] | 1.46 [0.99, 2.15] | 0.05 |
| Latine or Hispanic | 0.59 [0.26, 0.93] | 1.81 [1.30, 2.52] | 0.0005 |
| Age (>25 years old) | -0.35 [-0.61, -0.08] | 0.71 [0.54, 0.93] | 0.01 |

**Table S10.4. Boycotting**. Parameter estimates, standard error, and odds ratios for the effects of community cultural wealth, environmental education and other variables on participation in boycotting. Values between brackets correspond to 95% confidence intervals.

| **Variables** | **Estimate** | **Odds ratio** | **p-value** |
| --- | --- | --- | --- |
| Social Capital | 0.19 [0.06, 0.31] | 1.20 [1.06, 1.36] | 0.001 |
| Navigational Capital | 0.06 [-0.06, 0.18] | 1.06 [0.94, 1.19] | 0.34 |
| Resistant Capital | 0.50 [0.39, 0.61] | 1.65 [1.47, 1.84] | <0.0001 |
| Familial Capital | 0.12 [0.00, 0.25] | 1.13 [1.00, 1.29] | 0.06 |
| Black or African American | -0.80 [-1.15, -0.46] | 0.45 [0.32, 0.63] | 0.004 |
| Asian or Pacific Islander | -0.23 [-0.62, 0.15] | 0.79 [0.54, 1.16] | 0.24 |
| Latine or Hispanic | -0.20 [-0.53, 0.12] | 0.82 [0.59, 1.13] | 0.22 |
| Age (>25 years old) | -0.21 [-0.48, 0.06] | 0.81 [0.62, 1.06] | 0.12 |

**Table S10.5. Buycotting**. Parameter estimates, standard error, and odds ratios (OR) for the effects of community cultural wealth, environmental education and other variables on “buycotting”. Values between brackets correspond to 95% confidence intervals.

| **Variables** | **Estimate** | **Odds ratio** | **p-value** |
| --- | --- | --- | --- |
| Social Capital | 0.20 [0.07, 0.33] | 1.23 [1.08, 1.40] | 0.002 |
| Navigational Capital | 0.04 [-0.08, 0.17] | 1.05 [0.92, 1.18] | 0.48 |
| Resistant Capital | 0.47 [0.35, 0.59] | 1.60 [1.42, 1.80] | <0.0001 |
| Familial Capital | 0.10 [-0.04, 0.23] | 1.10 [0.96, 1.26] | 0.16 |
| Black or African American | -0.82 [-1.18, -0.47] | 0.44 [0.31, 0.63] | <0.0001 |
| Asian or Pacific Islander | -0.16 [-0.56, 0.23] | 0.85 [0.57, 1.26] | 0.41 |
| Latine or Hispanic | -0.03 [-0.37, 0.30] | 0.97 [0.69, 1.35] | 0.84 |
| Age (>25 years old) | -0.23 [-0.51, 0.06] | 0.79 [0.60, 1.06] | 0.11 |

**Table S10.6. Volunteering**. Parameter estimates, standard error (SE), and odds ratios (OR) for the effects of community cultural wealth, environmental education and other variables on volunteering for environmental organizations. Values between brackets correspond to 95% confidence intervals.

| **Variables** | **Estimate** | **Odds ratio** | **p-value** |
| --- | --- | --- | --- |
| Social Capital | 0.02 [-0.10, 0.15] | 1.02 [0.90, 1.16] | 0.73 |
| Navigational Capital | 0.11 [-0.01, 0.23] | 1.12 [0.99, 1.26] | 0.07 |
| Resistant Capital | 0.28 [0.17, 0.39] | 1.32 [1.19, 1.48] | <0.0001 |
| Familial Capital | 0.31 [0.18, 0.44] | 1.36 [1.20, 1.56] | <0.0001 |
| Black or African American | -0.52 [-0.87, -0.18] | 0.59 [0.42, 0.84] | 0.003 |
| Asian or Pacific Islander | -0.13 [-0.51, 0.26] | 0.88 [0.60, 1.30] | 0.52 |
| Latine or Hispanic | 0.28 [-0.04, 0.60] | 1.32 [0.96, 1.83] | 0.09 |
| Age (>25 years old) | -0.36 [-0.63, -0.09] | 0.70 [0.53, 0.91] | 0.01 |
